# SpyTag-Engineered Photoconvertible *Chenopodium album* Water-Soluble-Chlorophyll-Binding Protein as a HER2-Targeting Contrast Agent for Photoacoustic Imaging with Ex-Vivo Animal Validation

**DOI:** 10.1021/cbmi.6c00039

**Published:** 2026-06-10

**Authors:** Hannah H. Kim, Victor T. C. Tsang, Bingxin Huang, Simon C. K. Chan, Terence T. W. Wong

**Affiliations:** † Translational and Advanced Bioimaging Laboratory, Department of Chemical and Biological Engineering, 58207 The Hong Kong University of Science and Technology, Hong Kong 999077, China; ‡ Research Center for Medical Imaging and Analysis, The Hong Kong University of Science and Technology, Hong Kong 999077, China

**Keywords:** photoacoustic imaging, contrast agent, water-soluble-chlorophyll-binding protein, photoconversion, deep-tissue molecular targeting, differential imaging

## Abstract

Photoacoustic (PA) imaging is a powerful noninvasive modality that combines optical contrast with ultrasonic resolution. However, its clinical translation remains hindered by the lack of biocompatible contrast agents with high sensitivity and specificity for targeted biomarker visualization. Therefore, we here report a photoconvertible contrast agent based on the Class I water-soluble chlorophyll-binding protein from *Chenopodium album* (CaWSCP), engineered with a SpyTag-SpyCatcher system for site-specific conjugation to human epidermal growth factor receptor 2 (HER2)-targeting affibodies. Upon illumination, CaWSCP exhibits a distinct spectral shift from 668 to 742 nm, enabling differential imaging that effectively eliminates background interference—a critical advantage for molecular imaging in complex biological environments. The engineered agent demonstrates high biocompatibility (>90% cell viability at concentrations approximately 30–60 fold above detectable PA signals), specific targeting of HER2-positive cancer cells, and robust PA signal generation with a linear concentration-response relationship. Ex vivo studies using a tissue phantom demonstrate that the differential imaging approach provides enhanced contrast against a blood-mimicking background compared to preconversion signals, even in the presence of strong hemoglobin absorption. This chemically engineered, photoconversion-based strategy establishes CaWSCP as a versatile molecular imaging probe for background-suppressed PA imaging in cancer detection and image-guided interventions.

## Graphical abstract

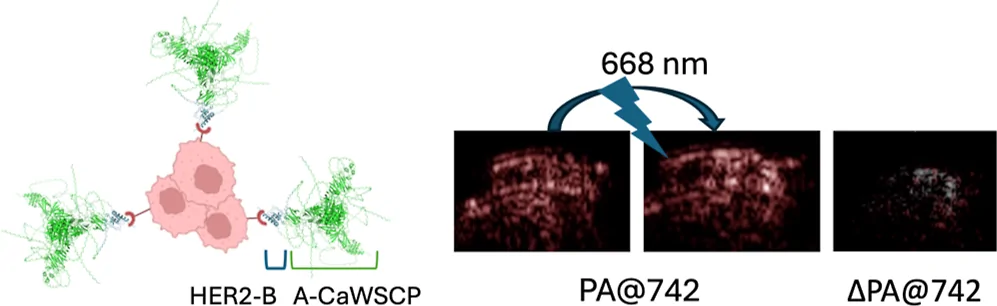

## 1 Introduction

Photoacoustic (PA) imaging has emerged as a powerful biomedical imaging modality, combining the high molecular contrast of optical imaging with the deep tissue penetration of ultrasound (US).^1,2^ By detecting acoustic waves generated from light absorption followed by thermal elastic expansion, PA imaging achieves multiscale resolution and molecular specificity without ionizing radiation, making it attractive for clinical applications such as cancer detection and vascular imaging.^3^ However, clinical translation is limited by the lack of biocompatible contrast agents with high sensitivity and specificity for targeted biomarkers at clinically relevant depths.^4^

Current PA contrast agents primarily include organic dyes and various nanoparticle formulations, each with significant limitations. Small molecule dyes, including U.S. Food and Drug Administration-approved indocyanine green (ICG), suffer from rapid clearance, poor photostability, and limited targeting capabilities, resulting in suboptimal contrast-to-noise ratios in complex biological environments.^5,6^ Even ICG, despite its clinical approval for certain applications, exhibits concentration-dependent aggregation, protein binding variability, and rapid degradation under physiological conditions, limiting its effectiveness for molecular PA imaging.^7^ Metallic nanoparticles (gold, silver, and carbon-based) generate strong PA signals but raise serious concerns regarding long-term toxicity, unpredictable biodistribution, and potential accumulation in vital organs.^8,9^ These drawbacks have hindered clinical translation despite promising preclinical results, highlighting the urgent need for alternative contrast strategies.^10^

In this regard, protein-based contrast agents are gaining attention across imaging modalities due to their inherent biocompatibility, structural versatility, and potential for molecular targeting.^11^ In magnetic resonance imaging and fluorescence imaging, protein-based agents have demonstrated improved relaxivity, extended circulation, and specific targeting with minimal background.^12,13^ Within the PA imaging field, genetically encoded proteins such as reversibly switchable fluorescent proteins have shown promise for background-free detection. However, these approaches require genetic modification of target tissues—an approach fraught with regulatory challenges and safety concerns for human applications.^14–17^ An ideal protein-based PA contrast agent would combine molecular targeting capability with intrinsic signal modulation while avoiding genetic manipulation of target tissues. Unlike conventional protein-based PA probes that primarily function as static absorbers, the proposed platform integrates a photoconvertible CaWSCP scaffold with HER2-targeted molecular recognition via affibody conjugation. This design enables differential imaging for background suppression while retaining the modularity and biocompatibility advantages of a protein-derived platform.

Among naturally occurring proteins with favorable optical properties, water-soluble chlorophyll-binding proteins (WSCPs) represent a particularly promising class of candidates for PA imaging applications. WSCPs efficiently absorb light through their porphyrin ring structure and convert it into heat, generating strong acoustic waves for high-resolution imaging.^18^ In their native state, WSCPs assemble into a tetrameric structure, with each subunit binding tightly to chlorophyll molecules in a specific arrangement, which stabilizes the chlorophyll–protein complex, optimizes light absorption, and makes energy conversion efficient.^19–21^ WSCPs offer additional advantages, including aqueous solubility and plant-derived biocompatibility. While previous work with *Lepidium virginicum* WSCP demonstrated feasibility as PA contrast agents,^22^ these first-generation agents lacked both molecular targeting capability and spectral modulation properties, limiting their specificity in complex biological environments.

Among WSCPs, the Class I WSCP from *Chenopodium album* (CaWSCP) is notable for its unique photoconversion: upon illumination, its absorption peak shifts from 668 to 742 nm while having all the other advantages of WSCPs.^23,24^ This spectral shift enables differential imaging, where pre- and post-illumination images can be spectrally unmixed to isolate the agent’s signal and eliminate background interference—a major advantage for deep tissue imaging that addresses the persistent challenge of distinguishing contrast agent signals from endogenous absorbers.^15^ The conventional PA imaging (Figure 1a) yields ambiguous tumor signals, while the chemically engineered CaWSCP probe (Figure 1b) enables HER2-targeted differential PA imaging via 668 → 742 nm photoconversion—a chemical tool for background-free molecular imaging.

**Figure 1:**
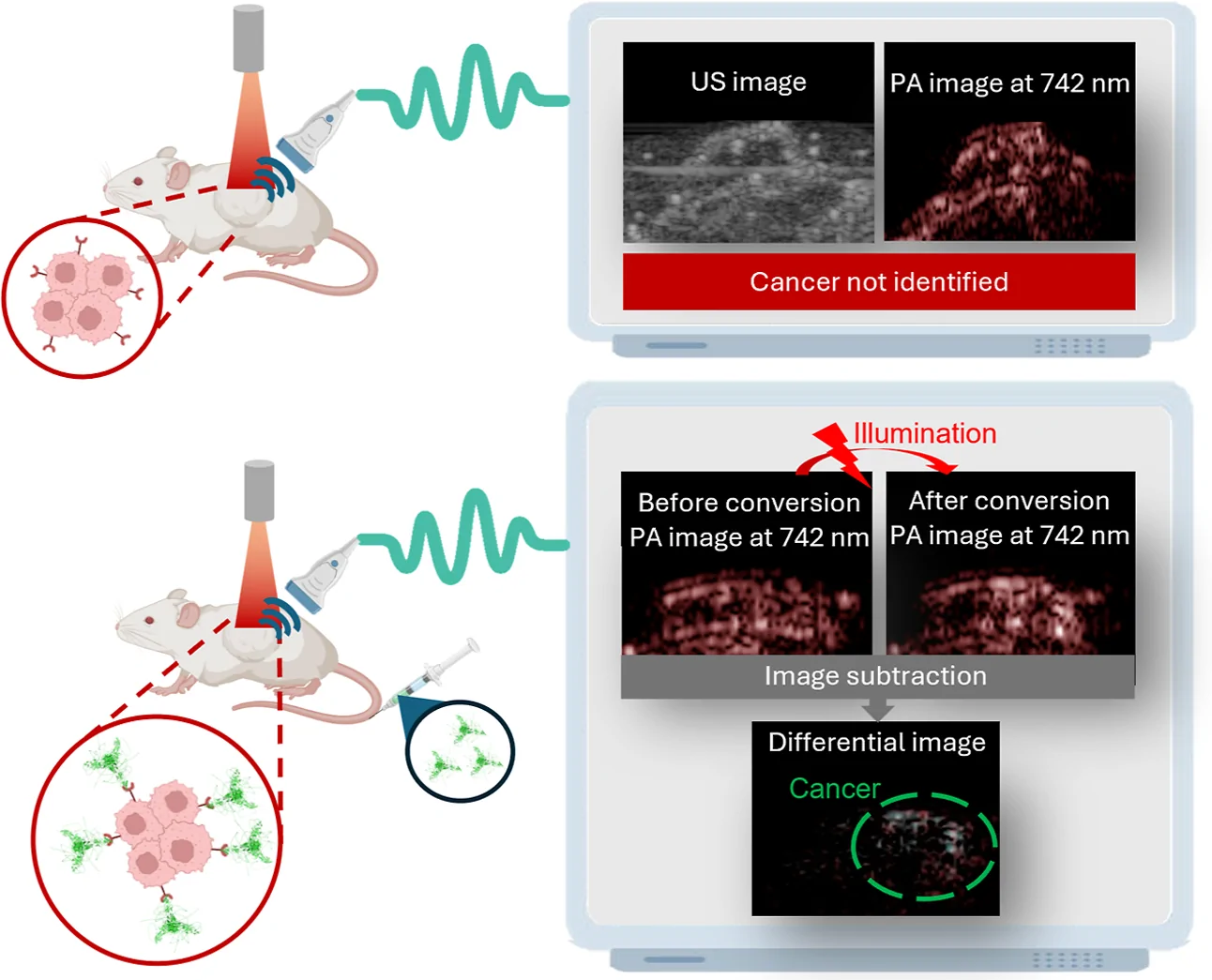
Differential PA imaging strategy using photoconvertible CaWSCP contrast agent for breast cancer detection. (a) Conventional PA imaging (even with US imaging mode) produces ambiguous signals with limited tumor contrast, preventing definitive cancer localization. (b) CaWSCP-enhanced approach utilizes targeted protein binding (green, inset) to cancer biomarkers. Differential imaging is achieved by subtracting preillumination from postillumination images at 742 nm, where signal enhancement occurs specifically at tumor sites following photoconversion (from 668 to 742 nm). This strategy effectively eliminates background interference, resulting in clear tumor delineation (yellow dotted line) with high molecular specificity.

To enhance molecular specificity, the SpyTag-SpyCatcher system was employed as a robust bioconjugation strategy enabling site-specific, covalent attachment of targeting moieties.^25,26^ By genetically encoding SpyTag onto CaWSCP and fusing SpyCatcher to targeting ligands, a modular platform adaptable to diverse molecular targets was created without compromising optical properties.

Human epidermal growth factor receptor 2 (HER2) was selected as a translationally relevant proof-of-concept biomarker due to its significant clinical role and overexpression in 20–30% of breast cancers, which is closely linked to poor prognosis.^27^ Importantly, this targeted imaging strategy is not inherently limited to HER2. The modular SpyTag-SpyCatcher conjugation design is highly versatile and can be readily adapted to diverse molecular targets by simply exchanging the affibody with alternative targeting ligands, peptides, or antibodies.

We report a photoconvertible CaWSCP-based PA contrast agent engineered via the SpyTag-SpyCatcher system and functionally conjugated to HER2-targeting affibodies. The agent enables selective imaging of HER2-positive cancer cells and exhibits favorable biocompatibility, targeting specificity, and strong PA signal in in vitro and phantom studies. Together, these results establish photoconvertible CaWSCP as a versatile platform for targeted PA imaging with potential impact on cancer detection, treatment monitoring, and image-guided interventions.

## 2 Results

### 2.1 Design and Characterization of Photoconvertible CaWSCP-Based Contrast Agent

The engineered contrast agent consists of CaWSCP modified with elastin-like polypeptide (ELP) linkers and SpyTag at both N- and C-termini (A-CaWSCP) for site-specific conjugation to HER2-targeting affibodies equipped with SpyCatcher (B-HER2). Sodium dodecyl sulfate-polyacrylamide gel electrophoresis (SDS-PAGE) analysis confirmed the successful modification of A-CaWSCP, showing the expected increase in molecular weight from approximately 21 kDa for unmodified CaWSCP (Figure 2a), (i) to 35 kDa for A-CaWSCP (Figure 2a), (ii). The successful conjugation of A-CaWSCP (Figure 2b), (iii) with B-HER2 affibody-SpyCatcher fusion protein that is around 20 kDa (Figure 2b), (iv) resulted in the formation of higher molecular weight bands corresponding to the CaWSCP-AB-HER2 complex at approximately 55 kDa (Figure 2b), (v). Further structural characterization of the protein complexes via size exclusion chromatography, transmission electron microscopy, and native PAGE confirmed the expected tetrameric assembly of A-CaWSCP (∼140 kDa) and its homogeneous particle distribution (∼10 nm diameter) (Supporting Information Figures S1–S3). These tetrameric structures maintained their integrity and spectroscopic properties when incubated in 50% FBS at 37 °C for up to 28 days, demonstrating robust stability under physiological conditions (Supporting Information Figure S4).

**Figure 2:**
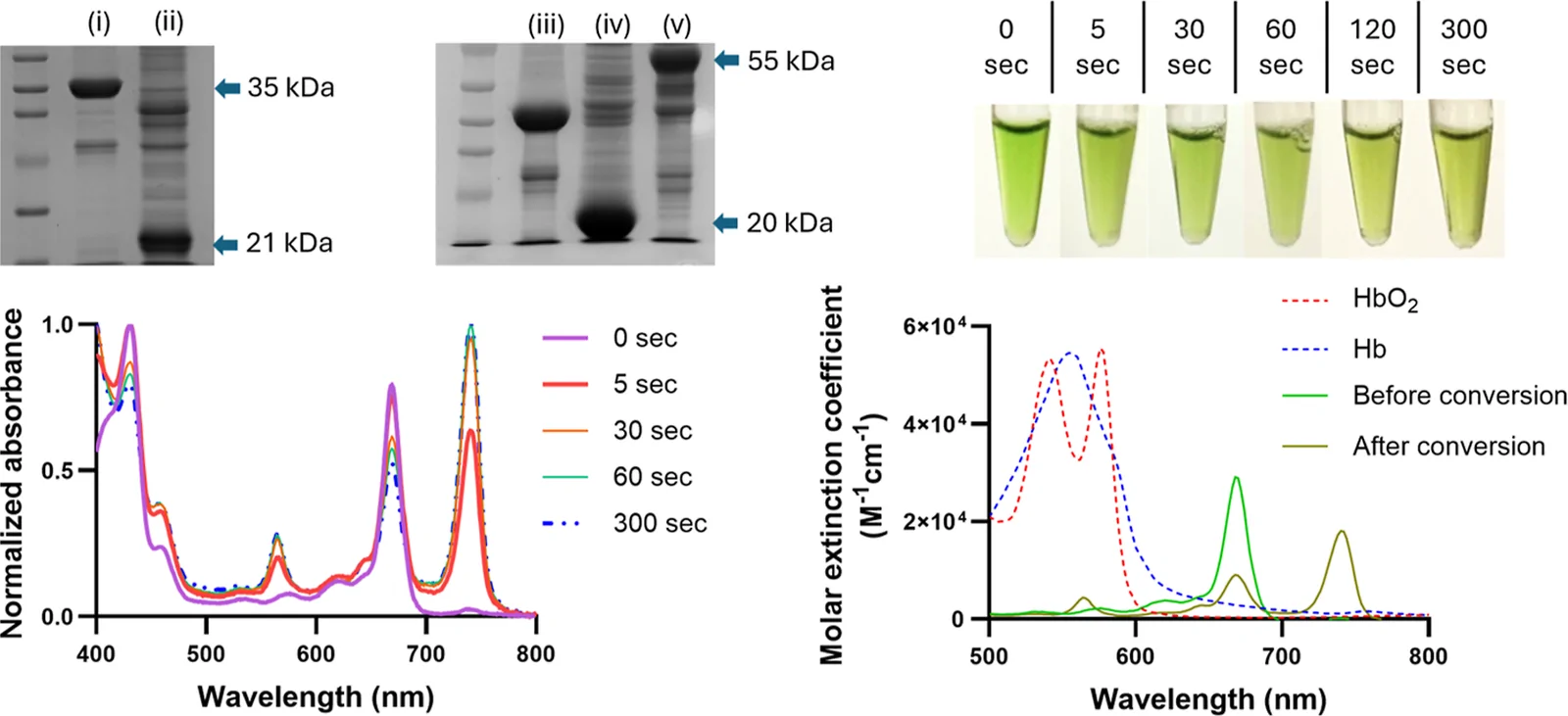
Spectroscopic and biochemical characterization of the engineered A-CaWSCP contrast agent. (a) SDS-PAGE analysis of unmodified (i) CaWSCP (21 kDa) and (ii) SpyTag-ELP-modified A-CaWSCP (35 kDa). (b) SDS-PAGE validation of (iii) A-CaWSCP (35 kDa) conjugation to (iv) B-HER2 (20 kDa), which form (v) CaWSCP-AB-HER2 complex (55 kDa). (c) Visual color changes of A-CaWSCP solution with increasing illumination duration (0–300 s). (d) Absorption spectra of A-CaWSCP before and after illumination at various time points. (e) Overlay of molar extinction coefficient spectra of A-CaWSCP before (green) and after conversion (brown), HbO_2_ (red), and Hb (blue).

Upon illumination, A-CaWSCP exhibited a visual color change from green to yellowish green with increasing illumination time (Figure 2c), corresponding to a significant red shift in its absorption spectrum from 668 to 742 nm (Figure 2d). The ratio of absorbance at 742 to 668 nm (A742/A668) increased progressively with illumination time, reaching a plateau after approximately 90 s without significant photobleaching (Supporting Information Figure S5). Consistent with retained structural integrity, far-UV circular dichroism (CD) spectra acquired before and after photoconversion displayed broadly similar secondary-structure signatures (Supporting Information Figure S6).

The molar extinction coefficients of A-CaWSCP before and after photoconversion are compared to those of oxyhemoglobin (HbO_2_) and deoxyhemoglobin (Hb) (Figure 2e). Before illumination, A-CaWSCP exhibited a stronger Qy absorption peak at approximately 668 nm, while after photoconversion, the primary peak shifted to approximately 742 nm, with the extinction coefficient remaining high in this spectral region.

Importantly, the photoconversion capability of A-CaWSCP remained robust across a wide range of physiologically relevant conditions. The protein maintained its photoconversion efficiency at temperatures ranging from 4 °C–50 °C (Figure 3a), across pH values from 5.0 to 8.0 (Figure 3b), and even when embedded within chicken breast tissue of varying thickness from 0 to 2 cm (Figure 3c), demonstrating potential for deep-tissue imaging applications. The corresponding absorbance signal was also retained under these conditions, further supporting the stability of the photoconverted state (Supporting Information Figure S7).

**Figure 3:**
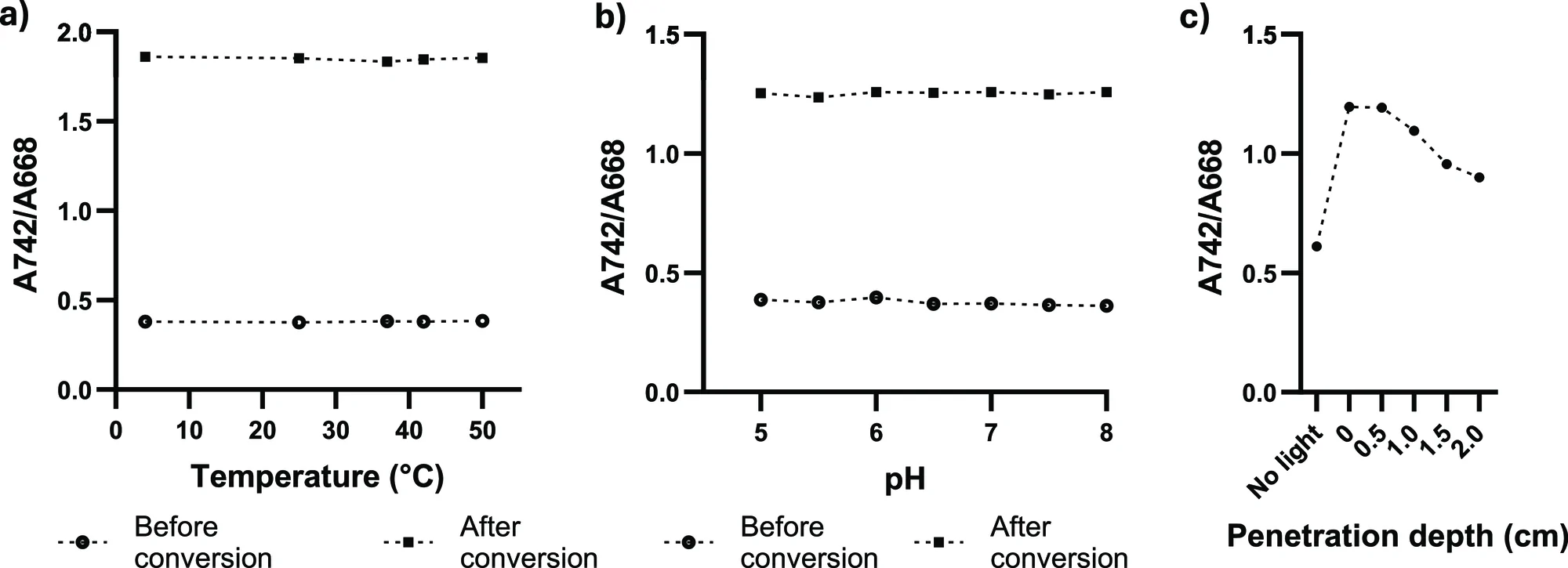
Photoconversion kinetics are shown as A742/A668 across different physiological conditions. (a) Temperature dependence (25–50 °C) of photoconversion efficiency. (b) pH dependence (pH 5.0–8.0) of photoconversion efficiency. (c) Photoconversion efficiency through increasing the thickness of chicken breast tissue.

### 2.2 Biocompatibility and Cell Viability Assessment

The biocompatibility of the contrast agent is crucial for clinical translation. MTT assays on 4T1 HER2-negative breast cancer cells demonstrated high cell viability (>90%) across concentrations of 0–150 μM and incubation times of 3–24 h, with no significant differences compared to controls (*p* > 0.05, one-way ANOVA with Dunnett’s post hoc test) (Figure 4a). At higher concentrations, cell viability remained above 90% up to 300 μM but decreased significantly at 400 μM and 500 μM (*p* < 0.001), establishing a clear upper bound for safe application (Figure 4b). The estimated concentration required for effective PA imaging (5–10 μM) provides a substantial safety margin of 30–60 fold.

**Figure 4:**
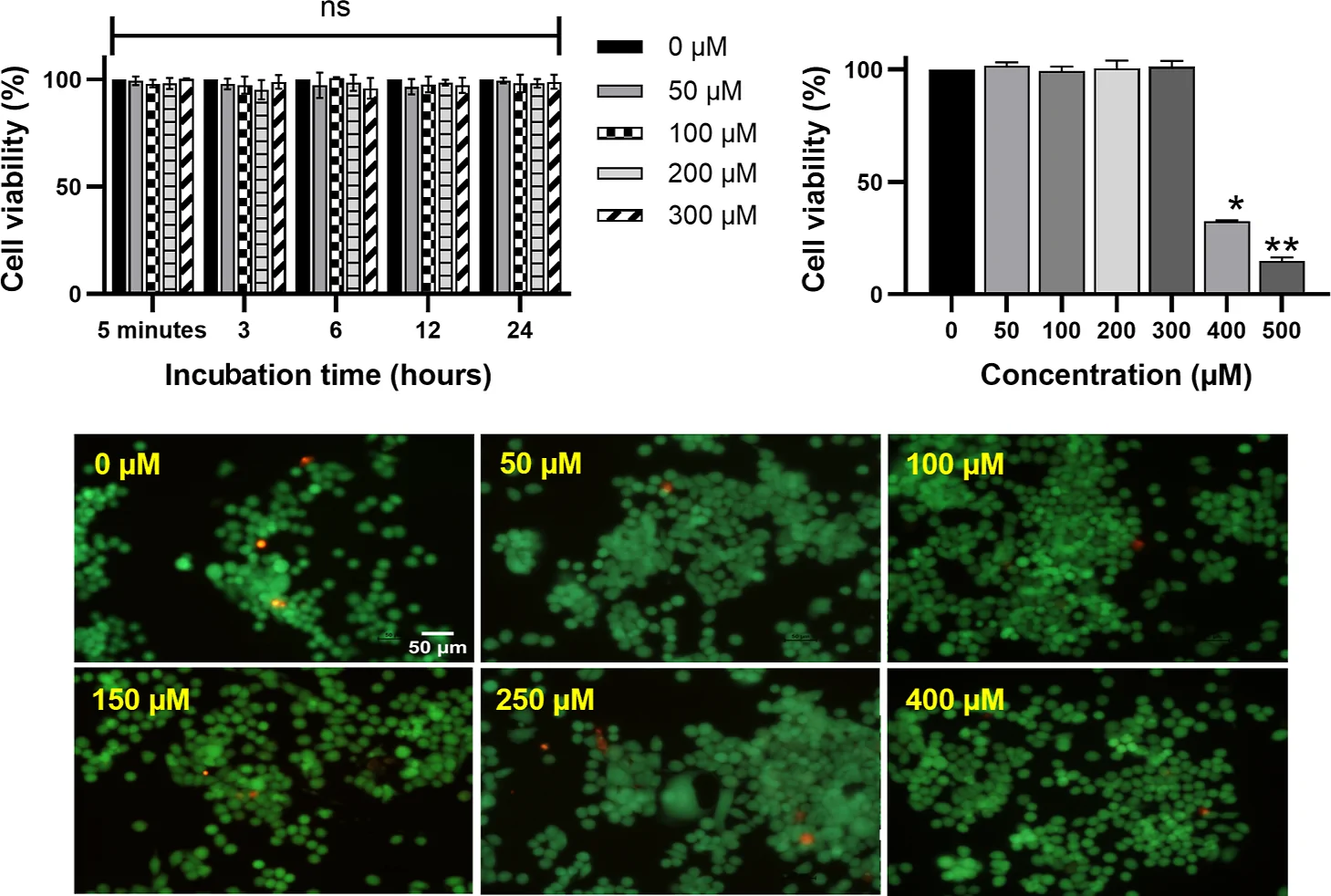
Biocompatibility evaluation of the engineered CaWSCP-AB-HER2 conjugate. (a) MTT assay of cell viability across different protein concentrations (0–150 μM) for various incubation periods (5 min, 3, 6, 12, and 24 h). Data represent mean ± standard deviation (SD) (*n* = 3). (b) MTT assay of concentration-dependent cell viability after 24 h incubation (0–500 μM). (c) Representative live/dead staining fluorescence microscopy images of cells after photoconversion (10 min illumination, laser at 668 nm, 10 mW/cm^2^) at varying protein concentrations (0–400 μM). Live cells appear green (calcein AM) and dead cells appear red (ethidium homodimer-1). Images representative of *n* = 3.

To assess phototoxicity, live/dead staining was performed on SKBR3 cells treated with CaWSCP-AB-HER2 and illuminated with a 668 nm laser for 10 min, exceeding the 90 s photoconversion time. Results showed minimal cytotoxicity at concentrations up to 300 μM, comparable to controls (Figure 4c). A slight increase in cell death was observed at 400 μM, consistent with MTT assay findings.

To further evaluate safety relevant to intravenous administration, hemocompatibility was assessed by a red blood cell hemolysis assay. CaWSCP-AB-HER2 demonstrated negligible hemolytic activity across all tested concentrations, with hemolysis values remaining close to 0% and within the range considered nonhemolytic for biomedical applications (Supporting Information Figure S8). These results, together with the MTT and phototoxicity data, collectively establish a broad safety profile that supports the suitability of CaWSCP-AB-HER2 for further in vivo investigation.

### 2.3 Molecular Targeting Capability

To quantify the binding affinity of CaWSCP-AB-HER2, a flow cytometry-based saturation-binding assay was performed on HER2-positive SKBR3 cells using increasing concentrations of the conjugate. The background-subtracted fluorescence intensities were analyzed as corrected mean fluorescence intensity (MFI) and fitted to the Hill equation by nonlinear regression. This analysis yielded a dissociation constant (*K*_d_) of 121.1 nM with a Hill coefficient of 2.332 (Figure 5a), indicating a saturable and concentration-dependent interaction between CaWSCP-AB-HER2 and HER2-expressing cells.

**Figure 5:**
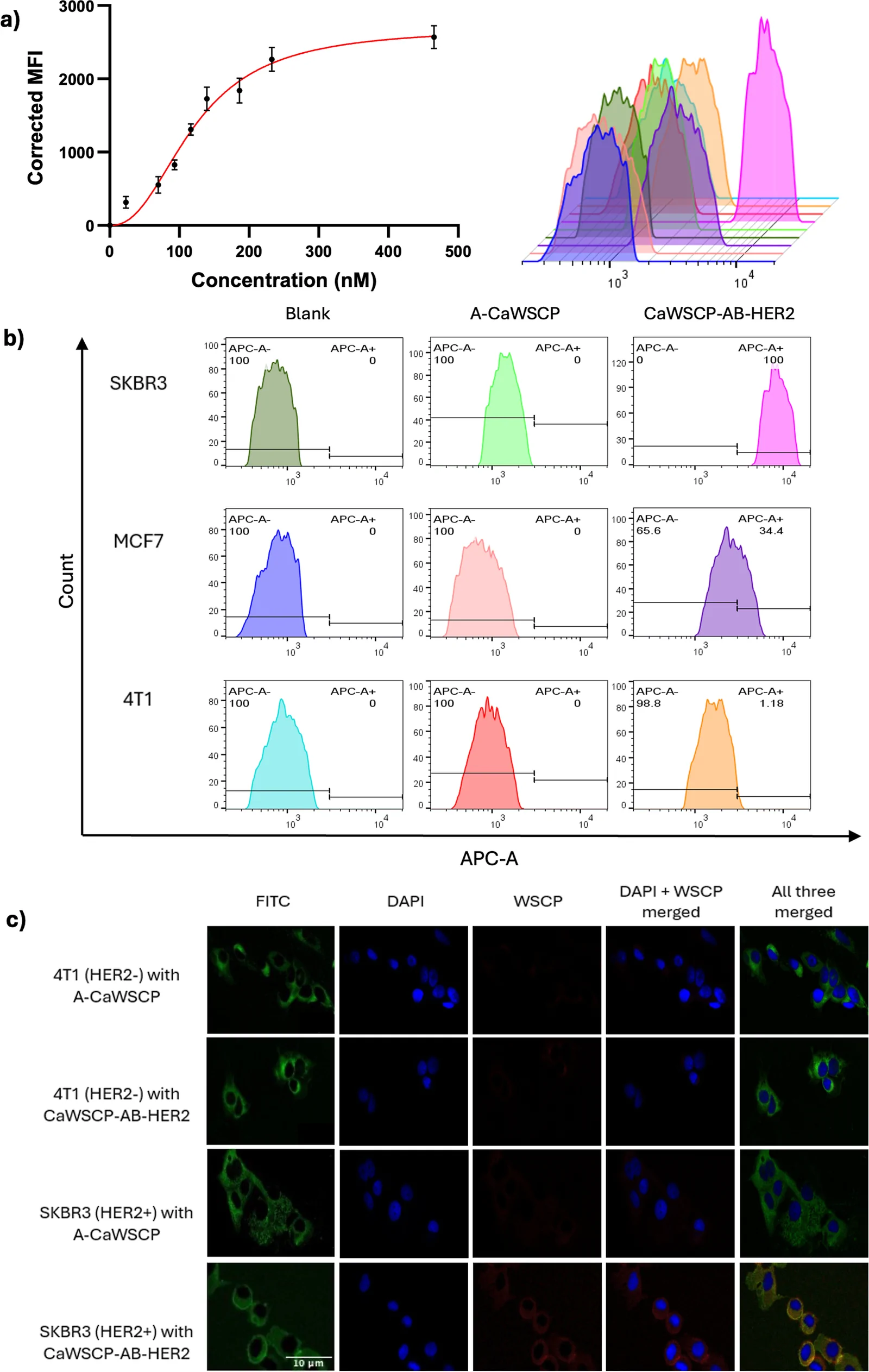
HER2-specific targeting and cellular binding of the engineered CaWSCP-AB-HER2 conjugate. (a) Flow cytometry-based saturation-binding analysis of CaWSCP-AB-HER2 on SKBR3 cells fitted to the Hill equation. (b) Flow cytometric analysis of selective binding using HER2-overexpressing SKBR3 cells, HER2-low-expressing MCF7 cells, and HER2-negative 4T1 cells. Data representative of *n* = 3 independent experiments. (c) Representative confocal microscopy images showing specific cellular binding in HER2-positive SKBR3 cells and no detectable binding in HER2-negative 4T1 cells. Images representative of *n* = 2.

To further assess HER2-dependent targeting across cell lines with different receptor expression levels, flow cytometric analysis was extended to include HER2-overexpressing SKBR3 cells, HER2-low-expressing MCF7 cells, and HER2-negative 4T1 cells. As shown in Figure 5b, CaWSCP-AB-HER2 produced the strongest right shift in fluorescence in SKBR3 cells, an intermediate shift in MCF7 cells, and minimal signal in 4T1 cells. In contrast, nontargeted A-CaWSCP and PBS controls showed little or no appreciable fluorescence across all three cell lines, confirming that the observed signal was driven by HER2-specific binding rather than nonspecific uptake. Supporting Information Figure S9 shows statistical differences.

Confocal microscopy further validated these findings by providing spatial information about the binding pattern. As shown in Figure 5c, CaWSCP-AB-HER2 demonstrated distinct membrane localization exclusively on HER2-positive SKBR3 cells (bottom row), with the red fluorescence signal (WSCP) colocalizing with the green CellMask membrane stain. No detectable red fluorescence was observed in any control conditions: HER2-negative 4T1 cells treated with CaWSCP-AB-HER2 (second row), or either cell line treated with nontargeted A-CaWSCP (first and third rows). The merged images show cell nuclei (blue), cell membranes (green), and the contrast agent (red), confirming specific binding at the membrane of HER2-positive cells. These results, consistent with flow cytometry data, demonstrate that the SpyTag-SpyCatcher conjugation strategy enables precise targeting of CaWSCP to HER2-expressing cancer cells. To further clarify the cellular distribution of the conjugate, supplementary internalization analysis showed that CaWSCP-AB-HER2 remained predominantly surface-associated under the tested conditions, consistent with the membrane-localized pattern observed by confocal microscopy (Supporting Information Figure S10).

Together, these results demonstrate that the SpyTag-SpyCatcher conjugation strategy preserves the targeting function of the HER2-affibody and enables specific, receptor-dependent binding of CaWSCP-AB-HER2 to HER2-expressing cancer cells.

### 2.4 PA Imaging Performance

For imaging experiments, a dual modality PA/US imaging system that incorporates an optical parametric oscillator (OPO) laser (repetition rate = 10 Hz, and pulse width = 5 ns) with tunable wavelength (335–2600 nm) was built (Figure 6a,b). This setup, an extension of the system described in previous work with *L. virginicum* WSCP, employs a commercial data acquisition system (DAQ) called Acoustic-X from Cyberdyne Inc., Tsukuba, Japan (Figure 6c).^22^ The system featured a specialized optical fiber bundle for efficient light delivery, a height-adjustable rotation stage for phantom positioning, and custom-designed signal processing algorithms for real-time image reconstruction. The US transducer had a 10 MHz center frequency with a −6 dB bandwidth of 80%, providing axial resolution of approximately 150 μm and lateral resolution of 450 μm at a depth of 10 mm. This allows for coregistered US/PA imaging, where US provides anatomical context while PA reveals molecular information from the contrast agent.

**Figure 6:**
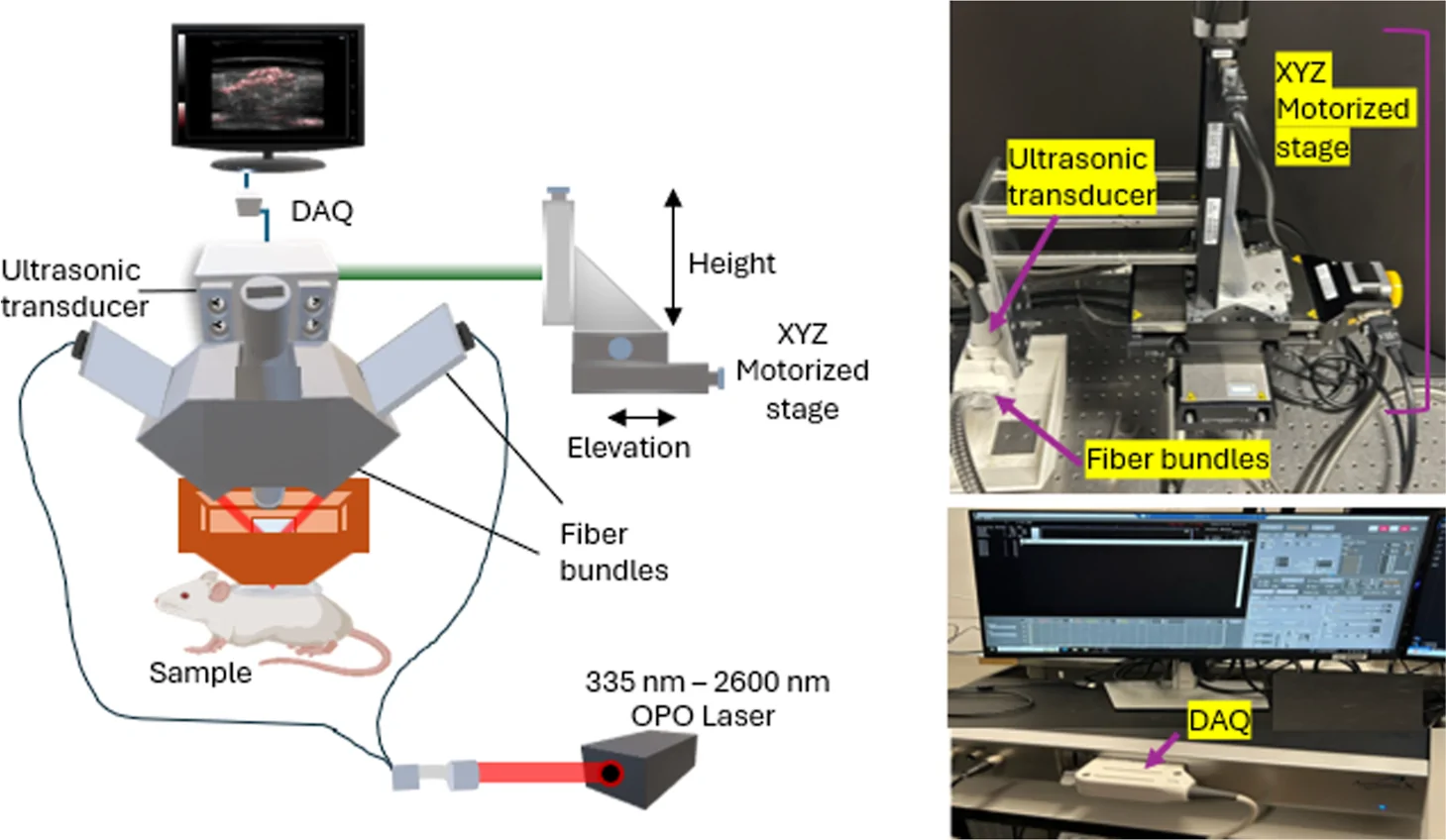
Dual-modality PA/US imaging system for contrast agent characterization. (a) Schematic illustration of our custom-built PA/US imaging system based on Acoustic-X. (b) A representative photograph of the actual experimental setup. (c) Detailed view of the Acoustic-X data acquisition system interface with 12 bit digitization at 40 MS/s sampling rate.

Phantom imaging experiments (Figure 7a) demonstrated clear visualization of the contrast agent at different concentrations. The dual-modality US/PA imaging shows both US structural information (top row) and PA signal (bottom row) across five samples with decreasing concentrations of CaWSCP conjugates (columns 1–5). The PA signal visibly decreased with lower concentrations, while maintaining clear detection even at the lowest concentration tested. The PA signal of CaWSCP conjugates exhibits a strong linear correlation with concentration (*R* ^2^ = 0.9882) over the range of 1–15 mg/mL (Figure 7b), indicating reliable quantitative imaging potential. This linear relationship is specifically observed at 742 nm (postphotoconversion, spheres), while minimal signal is detected at 762 nm (control wavelength, triangles), demonstrating the wavelength specificity of the photoconverted contrast agent. Additional characterization of photoconversion kinetics in relation to PA signal generation is provided in Supporting Information Figure S11.

**Figure 7:**
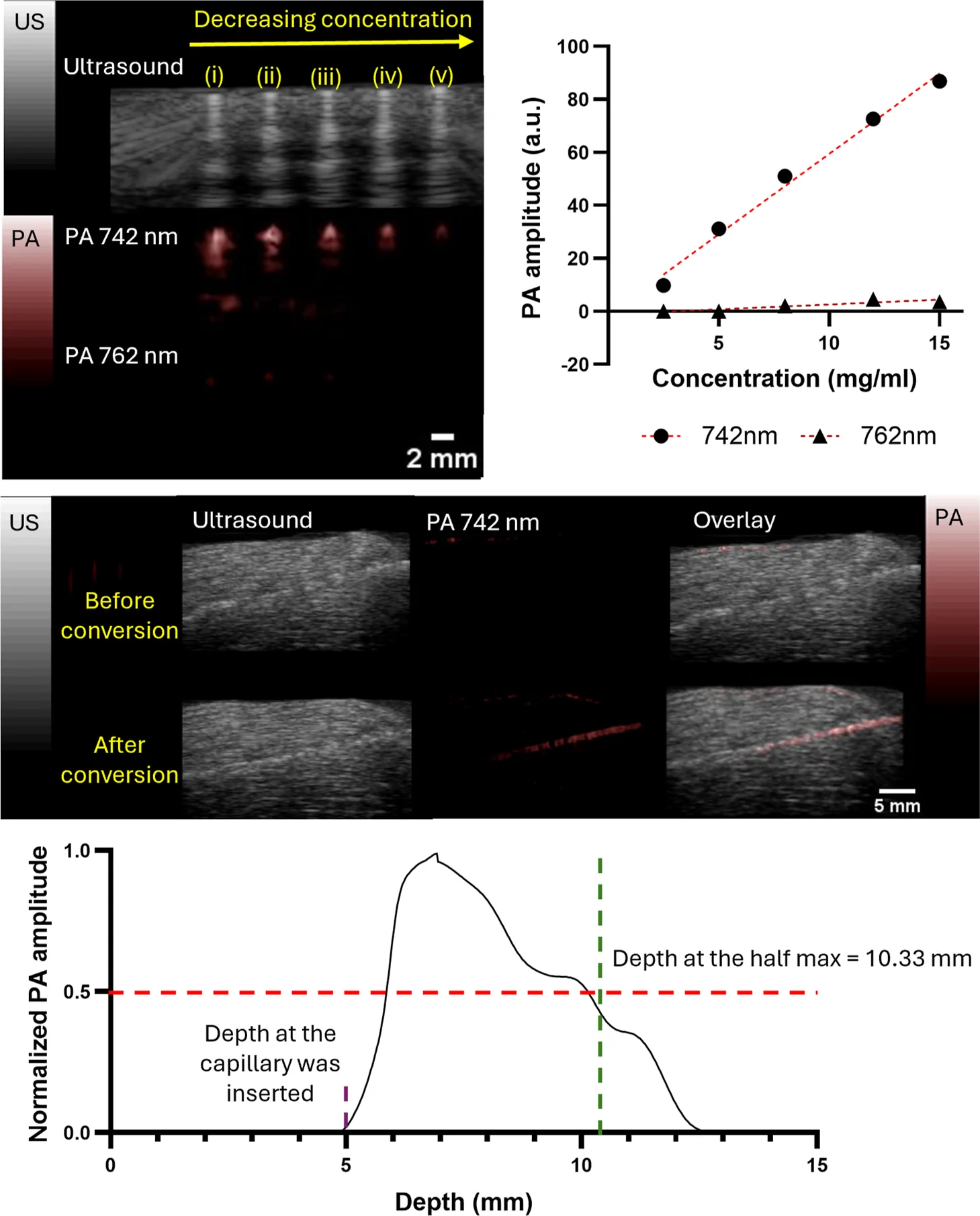
Image characterization of CaWSCP-AB-HER2 in tissue-mimicking phantoms. (a) US/PA signal measurements at different CaWSCP-AB-HER2 concentrations. (i: 15 mg/mL, ii: 10 mg/mL, iii: 5 mg/mL, iv: 2.5 mg/mL, v: 1 mg/mL). (b) Linear regression analysis of PA amplitude response at different concentrations at 742 nm and at a control wavelength 762 nm before and after photoconversion (*R* ^2^ = 0.9882). (c) US/PA imaging of CaWSCP-AB-HER2 embedded in chicken breast tissue before and after photoconversion. (d) Quantitative analysis of PA signal strength versus penetration depth from the tissue surface.

To evaluate performance in biological tissue, the contrast agent was embedded within chicken breast tissue at varying depths (Figure 7c). The comparison between prephotoconversion (left) and postphotoconversion (right) images showed enhanced PA signal (red) after photoconversion, with the signal clearly visible through the surrounding tissue. Notably, CaWSCP-AB-HER2 generated detectable PA signals even when positioned beneath 10.00 mm of tissue, demonstrating its potential for deep-tissue imaging applications. Quantitative analysis of signal attenuation with depth (Figure 7d) revealed that the PA signal strength decreased exponentially but reached its full-width at half-maximum (horizontal red dashed line) up to 10.33 mm beneath the tissue surface (vertical green dashed line), establishing the practical imaging depth limit for this contrast agent.

### 2.5 Ex Vivo Validation

To evaluate the performance of the contrast agent in a complex biological environment, ex vivo studies were conducted using mouse skin overlaid on gelatin phantoms (Figure 8a,b). The differential imaging capability was tested in the presence of blood, which generates strong PA signals that can mask contrast agent detection.

**Figure 8:**
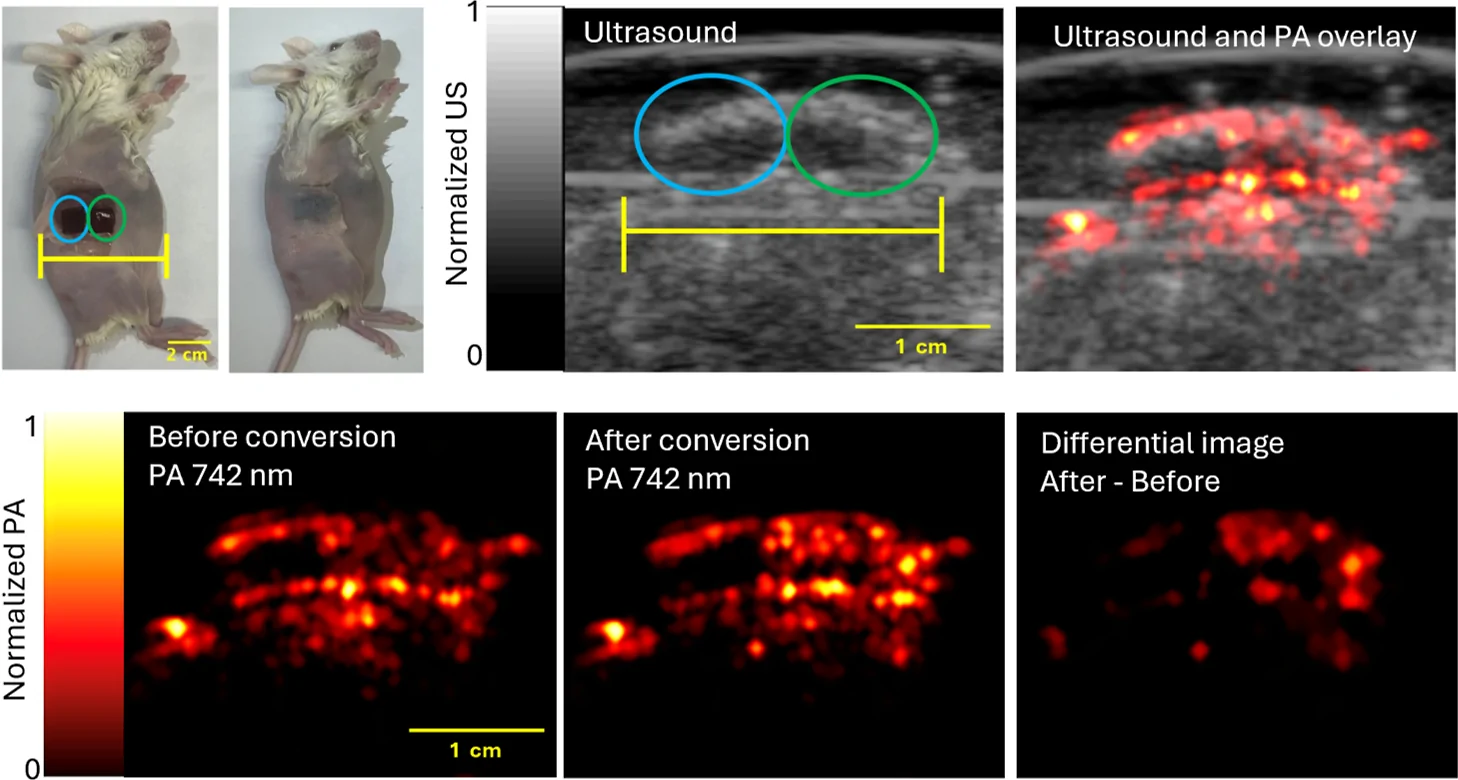
Ex vivo differential PA imaging validation in tissue-mimicking phantoms. (a) Photograph of gelatin phantoms with a blue circle indicating the blood-containing region and a green circle indicating the blood- and protein-containing region. (b) Photograph of a mouse with skin sealed back. (c) US image showing the structural information on phantoms in (a). (d) Combined US and PA image before photoconversion. (e) PA-only image before photoconversion. (f) PA-only image after photoconversion. (g) Differential PA image between (e,f).

The phantom contained a region with blood alone and a region with both blood and CaWSCP-AB-HER2. The US image provided anatomical context, while the prephotoconversion PA image showed strong signals from both regions due to blood absorption (Figure 8c,d). After photoconversion illumination, the postconversion PA image revealed a specific increase in PA signal in the region containing both blood and contrast agent. The differential PA image successfully isolated the signal from photoconverted CaWSCP-AB-HER2 while suppressing the blood background. The spectral properties of blood-containing gelatin phantoms were characterized by absorption spectroscopy to understand the optical interactions between the contrast agent and blood components (Supporting Information Figure S12). The absorption spectra revealed distinct spectral differences between blood alone and the mixture of blood with CaWSCP-AB-HER2, particularly after photoconversion. These spectral measurements provided the physical basis for the observed differential contrast enhancement in the PA images.

Qualitative contrast analysis was performed on all image types using Weber contrast, *C*_w_, defined as^28^

$$C_{w}=\frac{S-S_{b}}{S_{b}}$$

where *S* is the signal of interest and *S*_b_ is the background signal within the same image. The interstitial domain beneath the mouse skin (indicated by yellow lines in Figure 8c) served as the background region of interest (ROI). In the before photoconversion image (Figure 8e), both blood and blood + protein regions showed low Weber contrast values of 0.117 ± 0.012 and 0.107 ± 0.016, respectively, indicating poor contrast against the background and difficulty in distinguishing the contrast agent from blood. After photoconversion (Figure 8f), the blood region remained near baseline with the Weber contrast value of 0.131 ± 0.010 while the blood + protein region increased to a Weber contrast value of 0.654 ± 0.021, demonstrating an improvement in contrast between the two regions, although the contrast improvement remained limited. Most importantly, in the differential PA images (Figure 8g), the background was effectively canceled; therefore, the mean differential PA amplitude within the blood + protein ROI was robustly positive (27.72 ± 0.64) while the blood-only region remained close to the baseline (2.96 ± 0.17).

The analysis of all image types demonstrates that the differential imaging approach, which accounts for changes in PA signal due to photoconversion, provides the most effective contrast enhancement, yielding a near-zero-background subtraction image that isolates the agent’s photoconversion signal. This suggests that the photoconversion of CaWSCP-AB-HER2 is crucial for achieving specific contrast in complex biological environments, such as those containing blood.

Additional details and initial evaluations of the contrast agent in simpler phantom setups, such as comparisons with PBS, are provided in the Supporting Information Figure S13 to further support the findings presented.

## 3 Discussion

The photoconvertible CaWSCP-based contrast agent developed in this study represents a promising proof-of-concept for advancing molecular PA imaging through the combination of targeted delivery and dynamic optical properties. The successful engineering of CaWSCP with the SpyTag-SpyCatcher system resulted in a well-defined contrast agent with precise molecular architecture. The covalent isopeptide bond formed between these domains ensures stable attachment under physiological conditions, addressing stability concerns associated with antibody-based targeting approaches. This modular design allows for adaptation to various molecular targets without requiring a complete redesign of the contrast agent.

The photoconversion properties of the engineered protein CaWSCP represent a key innovation in this study. To contextualize the performance of CaWSCP-AB-HER2 relative to existing photoconvertible PA contrast agents, photoconversion itself is not a new concept in PA imaging, as Yao et al. previously demonstrated genetically encoded photoconvertible systems based on bacterial phytochromes. Building on this foundation, existing photoconvertible contrast agents are largely based on genetically encoded photochromic proteins, including engineered bacteriophytochromes and reversibly switchable fluorescent proteins that enable dynamic signal modulation and background suppression.^15,17^ While these approaches have improved photoconversion contrast and enabled advanced imaging functionalities, they remain dependent on genetic encoding or biologically mediated delivery, limiting their direct use as externally administered molecular imaging agents.^14^ In contrast, conventional small-molecule dyes and nanoparticle-based contrast agents, although not limited by genetic encoding, present distinct trade-offs. For example, ICG offers strong near-infrared absorption but is limited by rapid clearance and a lack of intrinsic targeting capability, while gold nanoparticles provide high PA signals and tunable optical properties yet raise concerns regarding inconsistent production, limited long-term biocompatibility, and uncertain clearance.

In comparison, the CaWSCP-AB-HER2 combines photoconversion-based signal differentiation with modular targeting in an externally administered format. The molecular targeting capability demonstrated through flow cytometry and confocal microscopy confirms the specificity of CaWSCP-AB-HER2 for HER2-expressing cells in vitro. The membrane-restricted distribution observed is particularly advantageous for PA imaging applications, as it maximizes accessibility to illumination light and may enhance photoconversion efficiency. In addition, the distinct spectral shift from 668 to 742 nm and the ability of CaWSCP to maintain structural and photoconversion stability under physiologically relevant conditions (pH 5.0–8.0, 4–50 °C, and through tissue up to 2 cm thick) support its performance in complex biological environments. The observed plateau in the A742/A668 ratio within ∼90 s further suggests that photoconversion reaches a near-complete spectral transition under these conditions, supporting efficient signal modulation for imaging applications. The achieved imaging depth (∼10 mm) further supports its applicability in clinically relevant scenarios. Furthermore, CaWSCP-AB-HER2 shows favorable biocompatibility, with high cell viability maintained even at concentrations far exceeding those required for imaging.^29^ Together, these features position CaWSCP-AB-HER2 as a complementary and translationally relevant alternative to existing photoconvertible PA contrast agents.

Beyond molecular design and targeting, the imaging performance of the platform further highlights its practical advantages. The PA performance in phantom and ex vivo studies demonstrates several advantages over current state-of-the-art contrast agents in three critical areas. First, the strong linear correlation between PA signal and concentration (*R* ^2^ = 0.9882) enables reliable quantitative imaging—a feature valuable for monitoring disease progression or treatment response. Second, the depth capability of 10.33 mm compares favorably to the typical 5–7 mm penetration achieved by conventional molecular contrast agents in similar tissue environments, expanding potential applications to detect deeper-seated lesions.^30^ Third, and perhaps most importantly, our differential imaging effectively separates the agent from the hemoglobin background. Rather than relying on the intrinsic molecular contrast from a single frame, which can be dominated by endogenous absorbers,^5,31,32^ we use photoconversion-based subtraction at 742 nm so that quasi-static absorbers cancel and the residual reports the agent’s spectral switch. Therefore, we present the Weber contrast for pre/post frames, and differential PA amplitude for the subtraction image to highlight agent-isolated signal on a near-zero-background baseline. This background-suppressed readout directly addresses a persistent challenge in molecular PA imaging—distinguishing specific molecular targets against the strong background of endogenous absorbers like hemoglobin. Additionally, A-CaWSCP exhibits environmental robustness rarely achieved with small-molecule dyes that often suffer from sensitivity to pH variations and rapid photobleaching. Based on the observed HER2-specific targeting, low cytotoxicity profile, and retained photoconversion behavior under physiologically relevant conditions (including temperature and pH variations), the CaWSCP-based platform demonstrates strong potential for in vivo molecular imaging. The ability to generate detectable, background-suppressed PA signals through tissue-mimicking media further supports the expectation that this differential imaging strategy will translate effectively to living systems. However, actual in vivo performance will naturally depend on additional physiological factors not addressed in the current proof-of-concept study. We also note that the use of controlled phantoms and simplified ex vivo tissue models is consistent with early stage validation strategies commonly employed in the PA imaging field to establish baseline performance prior to more complex in vivo studies. Key considerations for future clinical translations will include the agent’s circulation half-life, systemic biodistribution, background clearance rates, and the complex structural and vascular heterogeneity of the tumor microenvironment. Nevertheless, the protein-based scaffold and high-affinity affibody targeting are specifically designed to mitigate these issues compared to traditional small-molecule dyes. Therefore, while comprehensive in vivo studies are ultimately required for further evaluation, these rational design features are expected to enhance the probe’s performance in living systems.

These performance characteristics establish a strong foundation for future in vivo applications. The molecular size of the CaWSCP-AB-HER2 complex (∼150 kDa) represents an optimal balance for in vivo pharmacokinetics compared to existing contrast agents. While small molecule dyes like ICG (∼775 Da) suffer from rapid clearance (minutes) and poor target specificity, and large nanoparticles (>200 nm) face limited tumor penetration and potential long-term toxicity concerns, our agent occupies a favorable middle ground.^4^ At ∼150 kDa, CaWSCP-AB-HER2 is large enough to avoid rapid renal clearance (which typically occurs for molecules <40 kDa) yet small enough to potentially allow eventual elimination, unlike larger nanoparticles that often accumulate indefinitely in the reticuloendothelial system.^33,34^ Additionally, molecules in this size range have demonstrated favorable tumor penetration properties through the enhanced permeability and retention effect, while still maintaining sufficient circulation time for effective imaging.^35^ The ability to generate clear differential signals through mouse skin in our ex vivo model further suggests potential translatability to more complex in vivo environments, which is particularly encouraging for applications such as intraoperative margin assessment or lymph node mapping, where reliable signal detection through intervening tissues is essential for clinical utility. In addition to these advantages, the spectral properties of CaWSCP provide opportunities for further optimization. The current 668–742 nm spectral window is effective for differential imaging; however, future protein engineering of the chromophore-binding environment could enable tuning of the absorption profile further into the near-infrared region. Such adaptability represents an important avenue for extending the applicability of the CaWSCP scaffold to deeper tissue PA imaging systems.

Despite these promising results, several limitations must be acknowledged. First, while the current photoconversion system demonstrates robust spectral shifting within the 668–742 nm window, further optimization of the absorption profile toward deeper near-infrared regions may improve imaging depth and compatibility with clinical PA systems. During photoconversion, illumination is applied prior to image acquisition, and therefore does not interfere with subsequent signal detection. Future work should focus on improving photoconversion efficiency through protein engineering approaches. Additionally, the plant-derived nature of CaWSCP may present immunogenicity challenges for clinical translation, potentially requiring strategies to “humanize” the agent while preserving its photoconversion functionality. Most importantly, in vivo studies will be necessary to fully assess the pharmacokinetics, biodistribution, and imaging performance of the contrast agent in living subjects. In addition, the relatively large molecular size of the CaWSCP-AB-HER2 complex (∼150 kDa) may influence tissue penetration and diffusion in dense tumor microenvironments, which warrants further investigation.

Nevertheless, this proof-of-concept study demonstrates the potential of photoconvertible CaWSCP as a platform for molecular PA imaging with applications in cancer detection, treatment monitoring, and image-guided interventions. The combination of molecular targeting capability with intrinsic signal modulation addresses key limitations of existing contrast agents, potentially advancing PA imaging toward clinical translation. Future work will focus on in vivo validation and optimization of the photoconversion process to further evaluate the clinical potential of this technology.

## 4 Conclusion

A photoconvertible contrast agent based on CaWSCP has been developed for targeted PA imaging. Through engineering CaWSCP with the SpyTag-SpyCatcher system and conjugating it with HER2-targeting affibodies, a modular platform capable of selectively identifying HER2-positive cancer cells was created. The unique photoconversion properties of CaWSCP enable a differential imaging approach that effectively eliminates background interference, significantly enhancing contrast in complex biological environments. Comprehensive characterization demonstrates the agent’s excellent biocompatibility, targeting specificity, and PA performance across various conditions. These findings establish photoconvertible CaWSCP as a promising platform for molecular PA imaging with potential applications in cancer detection, treatment monitoring, and image-guided interventions. Future work will focus on in vivo validation and expansion to additional molecular targets, further advancing this technology toward clinical translation.

## 5 Experimental Section

### 5.1 Materials

General laboratory chemicals were obtained from Sigma-Aldrich unless otherwise specified. Cell culture reagents, including media and supplements, were sourced from Thermo Fisher Scientific. HER2-positive SKBR3 and HER2-negative 4T1 cell lines were purchased from ATCC.

### 5.2 Molecular Cloning

Genes encoding A-CaWSCP and B-HER2 were synthesized and cloned into pET22b­(+) vectors by GENEWIZ with codon optimization for *E. coli*. The A-CaWSCP sequence was based on the CaWSCP construct from Takahashi et al., with SpyTag (AHIVMVDAYKPTK) fused to both N- and C-termini^36^ (Supporting Information Figure S14). The B-HER2 construct consisted of a HER2-targeting affibody (ZHER2/342), fused to SpyCatcher via a (GGGGS)_3_ linker and an N-terminal His_6_-tag^37^ (Supporting Information Figure S15). All plasmids were sequence-verified before transformation into *E. coli* BL21­(DE3) for protein expression.

### 5.3 Protein Expression and Purification

*E. coli* BL21­(DE3) cells harboring pET22b/A-CaWSCP or B-HER2 were grown in LB with 100 mg/L ampicillin at 37 °C to OD600 0.6–1.0. Protein expression was induced with 0.4 mM IPTG for 3 h at 37 °C. Cells were harvested by centrifugation (4000*g*, 20 min, 4 °C) and resuspended in lysis buffer (50 mM Tris–HCl, 300 mM NaCl, 0.01% Triton X-100, pH 8.0).

For B-HER2, cells were lysed by sonication, centrifuged, and the supernatant purified by Ni-NTA chromatography (wash: 300 mM NaCl, 20 mM Tris, 25 mM imidazole, pH 7.5; elution: 300 mM NaCl, 20 mM Tris, 500 mM imidazole, pH 7.5). Proteins were dialyzed into PBS (pH 7.4) and concentrated using Amicon Ultra filters (10 kDa MWCO). Protein concentration was determined by NanoVue, and purity by SDS-PAGE.

A-CaWSCP required denaturation and refolding. Cells were lysed by sonication, and the lysate was treated with 8 M urea. The protein was dialyzed in three steps against refolding buffer (50 mM Tris–HCl, 150 mM NaCl, pH 8.0) with decreasing urea (4 M, 2 M, 0 M).

### 5.4 Chlorophyll Reconstitution

Chlorophyll reconstitution followed Takahashi et al.^36^ Spinach leaves (5× mass of *E. coli* pellet) were homogenized in 50 mM Tris–HCl, 100 mM NaCl, pH 8.0. The homogenate was centrifuged to collect thylakoid pellets. Dialyzed A-CaWSCP was added to the pellets, mixed, and incubated for 3 h at room temperature. After centrifugation, the supernatant was purified by Ni-NTA chromatography and buffer exchanged to remove imidazole. Purity was assessed by SDS-PAGE, and photoconversion was tested by measuring absorbance after room light exposure.

### 5.5 SpyTag-SpyCatcher Conjugation

Purified A-CaWSCP and B-HER2 were mixed at a 1:2 molar ratio in 50 mM Tris–HCl, 150 mM NaCl, pH 7.4, and incubated at 25 °C for 1 h. Conjugation was monitored by SDS-PAGE, with higher molecular weight bands indicating successful conjugation. The CaWSCP-AB-HER2 conjugate was purified by size exclusion chromatography (SEC).

Prior to developing the A-CaWSCP contrast agent, the SpyTag-SpyCatcher conjugation strategy was validated using enhanced green fluorescent protein (eGFP). An A-eGFP construct was generated by cloning the eGFP gene into the pET22b­(+) vector with SpyTag sequences at both N- and C-termini and verified via SDS-PAGE (Supporting Information Figure S16). Expression and purification of A-eGFP followed protocols identical to those described above, except without the chlorophyll reconstitution step. A-eGFP and B-HER2 were conjugated under the same conditions, and the resulting eGFP-AB-HER2 conjugates were purified by SEC.

To assess targeting specificity, HER2-positive SKBR3 and HER2-negative 4T1 cells were treated with PBS, A-eGFP (5 μM), or eGFP-AB-HER2 (5 μM) for 15 min at room temperature. Cells were washed, fixed, and imaged using a Nikon Eclipse Ti2 inverted research microscope. The specific binding of eGFP-AB-HER2 to HER2-positive SKBR3 cells, with no detectable binding to HER2-negative 4T1 cells, established the efficacy of the SpyTag-SpyCatcher targeting approach (Supporting Information Figure S17) before proceeding with the development of the photoconvertible contrast agent.

### 5.6 Protein Characterization

Protein purity and oligomeric state were assessed by SDS-PAGE, native PAGE, and SEC. SDS-PAGE was performed using 12.5% polyacrylamide gels with 4× Laemmli buffer (10% β-mercaptoethanol), heated at 95 °C for 5 min, and run at 120 V for 70 min. Gels were stained with Coomassie Brilliant Blue R-250 and destained in 40% methanol, 10% acetic acid. Molecular weights were estimated using a prestained ladder (10–250 kDa). Native PAGE was performed using 10% polyacrylamide gels at 4 °C, 80 V for 120 min, and stained with Coomassie Brilliant Blue R-250.

SEC was conducted using a Bio-Rad FPLC system equipped with an ENrich SEC 70 10 × 300 mm column (#7801070), equilibrated with 50 mM Tris–HCl, 150 mM NaCl, pH 7.4, at 0.5 mL/min. Protein samples (100 μL, 1–2 mg/mL) were injected, and elution was monitored at 280 nm (protein) and 668 nm (chlorophyll). Elution profiles were compared to molecular weight standards to confirm tetrameric assembly of A-CaWSCP (∼140 kDa) and to verify the oligomeric state of CaWSCP-AB-HER2 conjugates. Fractions were pooled, concentrated (30 kDa MWCO), and quantified by NanoDrop.

TEM analysis was performed to further confirm the homogeneity and tetrameric assembly of the protein complexes. Protein samples (5 μL, 0.1 mg/mL) were applied to glow-discharged carbon-coated copper grids and negatively stained with 2% uranyl acetate. Samples were imaged using a Thermo Scientific Talos120c microscope operated at 120 keV, equipped with a LaB6 filament and a charge-coupled device (CCD) detector. Images were collected at nominal magnifications of 45,000–92,000× with a pixel size of 0.24 nm. Particle size distribution analysis was performed using ImageJ software (NIH, version 1.53c) to verify the expected dimensions of tetrameric A-CaWSCP (∼10 nm diameter).

For serum stability testing, A-CaWSCP (200 μg/mL) was incubated in 50% FBS diluted in PBS at 37 °C for extended periods (1, 3, 7, 14, and 28 days). Samples were analyzed immediately at each time point by native PAGE and spectroscopic measurements. The persistence of the tetrameric band (∼140 kDa) and the A668/A280 ratio were monitored as indicators of structural stability. All experiments were performed in triplicate.

Far-UV CD measurements were performed to evaluate the secondary structure of A-CaWSCP before and after photoconversion. Protein samples were prepared in PBS at identical concentrations and measured using a quartz cuvette (0.1 cm path length) on a CD spectropolarimeter (J-815, Jasco, Japan). Spectra were recorded over the wavelength range of 195–260 nm at room temperature. Photoconversion was induced by illumination at 668 nm prior to acquisition of the “after” spectra. Buffer spectra were collected under identical conditions and subtracted from all measurements. The resulting CD signals are presented as ellipticity (mdeg) as a function of wavelength. The data is presented in Supporting Information Figure S6.

### 5.7 Spectroscopic Characterization

UV–vis spectra of A-CaWSCP and CaWSCP-AB-HER2 (100 μg/mL in PBS) were recorded in 96-well plates (200 μL/well) using a Varioskan Multimode reader (Thermo Fisher Scientific, VL0L0TD0), scanning 300–800 nm before and after illumination with a 668 nm light-emitting diode (LED) (10 mW/cm^2^) for 0–120 s. Photoconversion was quantified by the A742/A668 ratio. Fluorescence emission spectra (ex 400 nm, em 600–800 nm) were also recorded. All measurements were performed in triplicate at room temperature, with PBS as a blank.

Fluorescence emission measurements were performed as part of the standard characterization protocol, despite ideal PA contrast agents having minimal fluorescence. Using the same Varioskan Multimode reader, fluorescence spectra of A-CaWSCP and CaWSCP-AB-HER2 (100 μg/mL in PBS) were recorded in black 96-well plates (excitation: 400 nm, emission: 600–800 nm, 5 nm step size). These measurements confirmed sufficient signal for flow cytometry and confocal microscopy detection, while verifying that our contrast agent primarily converts absorbed energy to heat rather than light. Complete fluorescence emission spectra are presented in Supporting Information Figure S18.

### 5.8 Photoconversion Stability Test

The stability and efficiency of A-CaWSCP photoconversion were evaluated under various physiologically relevant conditions. All experiments were conducted under dark conditions except during controlled illumination periods, and photoconversion was quantified by measuring the ratio of absorbance at 742–668 nm (A742/A668) using a Varioskan Multimode reader (Thermo Fisher Scientific, VL0L0TD0). For all stability tests, samples were illuminated using the OPO laser system described in Section 5.12, tuned to 668 nm with consistent power (10 mW/cm^2^) for a standardized duration of 5 min to ensure comparable results across different experimental conditions. All experiments were performed in triplicate, with results presented as mean ± standard deviation. Temperature dependence was assessed by pre-equilibrating A-CaWSCP samples (100 μg/mL in PBS) at different temperatures (4 °C, 25 °C, 37 °C, and 50 °C) for 30 min in a temperature-controlled water bath before illumination. Samples were maintained at their target temperatures throughout the illumination period. pH stability was evaluated by preparing A-CaWSCP (100 μg/mL) in buffers ranging from pH 5.0 to 8.0 (50 mM sodium acetate for pH 5.0–5.5, 50 mM MES for pH 6.0–6.5, and 50 mM Tris–HCl for pH 7.0–8.0, all containing 150 mM NaCl). Samples were equilibrated in their respective buffers for 1 h at room temperature before illumination. Tissue penetration experiments were conducted by placing chicken breast tissue of varying thickness (0–2 cm) between the OPO laser source and A-CaWSCP samples (200 μg/mL in PBS) in a custom-designed holder. Absorbance spectra were measured immediately after illumination to determine the photoconversion efficiency as a function of tissue thickness.

### 5.9 Cell Viability Assays

SKBR3 and 4T1 cells were cultured in DMEM and RPMI, respectively, with 10% FBS, 100 U/mL penicillin, and 100 μg/mL streptomycin at 37 °C, 5% CO_2_. Cells were passaged at 80% confluence using 0.25% trypsin-EDTA.

Cell viability was assessed by MTT assay (Thermo Fisher Scientific). For dose-response, SKBR3 cells were seeded in 96-well plates (1 × 10^4^ cells/well), adhered overnight, and treated with CaWSCP-AB-HER2 (0–150 μM) or PBS for 3, 6, 12, or 24 h. For the toxicity threshold, cells were treated with 150 μM–1 mM CaWSCP-AB-HER2 for 24 h. After treatment, the medium was replaced with MTT (0.5 mg/mL), incubated for 3 h, and formazan dissolved in 100 μL DMSO. Absorbance was measured at 570 nm (ref 630 nm). Viability was calculated relative to controls. All experiments were in triplicate; results are mean ± SD.

For photoconversion cytotoxicity, SKBR3 cells were seeded in 6-well plates (2 × 10^5^ cells/well), adhered overnight, incubated with CaWSCP-AB-HER2 (50 μM) for 1 h, washed, and illuminated with a 668 nm LED (10 mW/cm^2^) for 10 min. Controls included nonilluminated and untreated cells. Cells were stained with Live/Dead Viability Kit (calcein AM 2 μM, ethidium homodimer-1 4 μM, 30 min, 37 °C) and imaged with a Nikon Eclipse Ti2 microscope.

Cell viability assays were performed in triplicate (*n* = 3) to ensure experimental reproducibility, with quantitative data presented as mean ± SD. Statistical significance was determined using one-way analysis of variance (ANOVA) followed by Dunnett’s posthoc test for multiple comparisons, with a *p*-value of <0.05 considered statistically significant.

### 5.10 Hemocompatibility Assay

Mouse blood was collected into EDTA-containing tubes and gently mixed immediately after collection. Red blood cells (RBCs) were isolated by centrifugation, washed three times with PBS, and resuspended as a 2% suspension in PBS. RBCs were incubated with CaWSCP-AB-HER2 at the indicated concentrations for 1 h at 37 °C. PBS and deionized water were used as negative and positive controls, respectively. Following incubation, samples were centrifuged, and the supernatants were transferred to a 96-well plate. Released hemoglobin was quantified by measuring absorbance at 540 nm using a Varioskan Multimode reader (Thermo Fisher Scientific, VL0L0TD0). Hemolysis (%) was calculated relative to the negative and positive controls.

### 5.11 Flow Cytometry and Binding Affinity Analyses

Flow cytometry was performed on a BD FACS Aria III. HER2 expression was confirmed by staining SKBR3 and 4T1 cells with Alexa Fluor 640-conjugated anti-HER2 antibody (1:100, 30 min, 4 °C), detected in the APC-A channel (633 nm ex.). The data is presented in Supporting Information Figure S19.

To determine the apparent binding affinity of CaWSCP-AB-HER2, a saturation binding assay was performed using HER2-positive SKBR3 cells. Cells were incubated with increasing concentrations of CaWSCP-AB-HER2 (0 nM, 23 nM, 70 nM, 93 nM, 116 nM, 140 nM, 187 nM, 233 nM, and 465 nM), and fluorescence was measured in the APC-A channel after background subtraction. Corrected mean fluorescence values were obtained from three independent experiments performed in triplicate and fitted to the Hill equation by nonlinear regression in GraphPad Prism to calculate the *K*_d_ and Hill coefficient.

For assessing the targeting specificity, cells were seeded in 6-well plates (5 × 10^6^ cells/well), treated with PBS, A-CaWSCP (5 μM), or CaWSCP-AB-HER2 (5 μM) for 30 min at room temperature, washed, and detached with 0.05% versene. Chlorophyll fluorescence was detected (633 nm ex, APC-A channel). At least 10,000 events were analyzed per sample using FlowJo v10.8, with gating to exclude debris and doublets. Mean fluorescence intensity quantified binding specificity. Mean fluorescence intensity was calculated for each condition across three independent experiments. Statistical analysis was performed using a two-tailed *t*-test.

All flow cytometry experiments were performed in triplicate (*n* = 3). Statistical analysis of cellular binding interactions was evaluated using fold differences in mean fluorescence intensity relative to controls. To quantify the magnitude of the targeting specificity, effect sizes were calculated using Cohen’s *d*.

### 5.12 Confocal Microscopy

SKBR3 and 4T1 cells were seeded on confocal dishes (5 × 10^4^ cells/well), cultured overnight, and treated with PBS, A-CaWSCP (5 μM), or CaWSCP-AB-HER2 (5 μM) for 15 min at room temperature. Cells were washed, fixed with 4% paraformaldehyde (15 min), stained with 4′,6-diamidino-2-phenylindole (DAPI) (1 μg/mL, 5 min), and CellMask Green (1:1000, 10 min). Confocal images were acquired using a Nikon C2 microscope (20× objective; DAPI: 405/415–480 nm, CellMask: 488/500–550 nm, chlorophyll: 633/650–750 nm). Co-localization was analyzed in ImageJ (JACoP plugin).

For 3D localization analysis, SKBR3 cells were treated with PBS (blank), A-CaWSCP, or CaWSCP-AB-HER2 (5 μM) for 1 h at 37 °C, followed by washing with PBS to remove unbound protein. Brightfield and Cy5 fluorescence images were acquired using the same confocal settings. *Z*-stack images were collected across the full cellular depth (99 slices), and 3D volume renderings, orthogonal views, and *xz* projections were reconstructed using ImageJ/Fiji (3D Viewer plugin). All images were processed using identical display settings. The data is presented in Supporting Information Figure S10.

### 5.13 PA Imaging and Phantom Studies

PA imaging used an OPO laser (335–2600 nm) focused at the sample position, coupled to a 10 MHz US transducer. Samples were held in glass capillaries (0.5 mm diameter) in a custom water tank, with positioning controlled by a motorized XYZ stage. PA signals were acquired and processed for real-time imaging.

For concentration studies, CaWSCP-AB-HER2 (1–15 mg/mL) was loaded into capillaries and imaged at 742 nm. All measurements were in triplicate. For photoconversion, PA signals were measured before and after illumination with a 668 nm light source at the focal point for 0–120 s. Photobleaching was assessed by continuous illumination at 668 nm up to 30 min, with the PA signal measured at intervals.

To ensure the reproducibility of the PA signal measurements, all image acquisitions were repeated across three independent phantom preparations (*n* = 3). Quantitative PA amplitudes were extracted from the defined ROIs and are reported as mean ± SD. Linear regression analysis was performed using GraphPad Prism to evaluate the concentration-dependent signal response.

### 5.14 Ex Vivo Validation

Ex vivo validation used mouse carcasses obtained from the HKUST Laboratory Animal Facility (LAF) under approved protocol AEP-2022-0084. The animals had been euthanized for humane reasons or at the end of their natural lifespan as part of other research protocols.

Gelatin phantoms (0.5 g/5 mL water) containing CaWSCP-AB-HER2 (5 mg/mL) or PBS were prepared and placed beneath excised mouse skin, secured with cyanoacrylate. In a second set, blood was added to mimic tissue background. For each condition, *n* = 3 independent replicates were performed. PA images were acquired at 742 nm before and after illumination. Averaging with 700 frames was employed to improve image quality, which is consistent with established PA imaging practices where the number of averaging typically ranges from 100–1000 frames depending on the applications.^30,32,38^

Differential imaging was performed in ImageJ (NIH, version 1.53c) by subtracting preillumination from postillumination images using the Image Calculator function. Weber contrasts were calculated by defining ROIs in both signal and background areas using the oval selection tool. For PBS phantoms, circular ROIs (diameter: 3 mm) were placed over the CaWSCP-AB-HER2-containing region (signal) and PBS-containing region (background). For blood-containing phantoms, ROIs were similarly placed over the region containing both blood and CaWSCP-AB-HER2 (signal) and the region containing only blood (background). The mean pixel value was measured for each ROI using the Measure function in ImageJ. Weber contrasts were calculated using the equation from Peli.^28^ For each phantom type, measurements were taken from three independent replicates and reported as mean ± SD.

## Supplementary Material



## Data Availability

The data supporting this study are available from the corresponding author upon reasonable request.

## Notes

Additional protein characterization, stability analyses, hemocompatibility assessment, cellular targeting validation, photoconversion-related PA analysis, ex vivo phantom validation, construct designs, amino acid sequences, and supplementary fluorescence and flow cytometry data (PDF)

## Abbreviations

PA: photoacoustic
US: ultrasound
ICG: indocyanine green
CaWSCP: *Chenopodium album* water-soluble chlorophyll-binding protein
HER2: human epidermal growth factor receptor 2
A-CaWSCP: CaWSCP modified with SpyTag
B-HER2: HER2-targeting affibody fused with SpyCatcher
CaWSCP-AB-HER2: A-CaWSCP and B-HER2 conjugates
ELP: elastin-like polypeptide
PBS: phosphate-buffered saline
SDS-PAGE: sodium dodecyl sulfate-polyacrylamide gel electrophoresis
HbO_2_: oxyhemoglobin
Hb: deoxyhemoglobin
MTT: 3-(4,5-dimethylthiazol-2-yl)-2,5-diphenyltetrazolium bromide
OPO: optical parametric oscillator
DMEM: Dulbecco’s modified eagle medium
RPMI: roswell park memorial institute medium
FBS: fetal bovine serum
DAPI: 4′,6-diamidino-2-phenylindole
SEC: size exclusion chromatography
ROI: region of interest
