# Supplementary material for: SpyTag-Engineered Photoconvertible Chenopodium album Water-Soluble-Chlorophyll-Binding Protein as a HER2-Targeting Contrast Agent for Photoacoustic Imaging with Ex-Vivo Animal Validation
Source: Chem Biomed Imaging. 2026 Jun 10;4(10):2246–59. doi: 10.1021/cbmi.6c00039 (PMC13715364; doi:10.1021/cbmi.6c00039)
Supplement: Supplementary file 1 [file im6c00039_si_001.pdf]

Supporting Information

SpyTag-Engineered Photoconvertible *Chenopodium album* Water-Soluble Chlorophyll-Binding Protein as a HER2-Targeting Contrast Agent for Photoacoustic Imaging with *Ex-vivo* Animal Validation

Hannah H. Kim[1], Victor T. C. Tsang[1], Bingxin Huang[1], Simon C. K. Chan[1], and Terence T. W. Wong[1,2,*]

[1]Translational and Advanced Bioimaging Laboratory, Department of Chemical and Biological Engineering, The Hong Kong University of Science and Technology, Hong Kong, China.
[2]Research Center for Medical Imaging and Analysis, The Hong Kong University of Science and Technology, Hong Kong, China.

*Correspondence: ttwwong@ust.hk

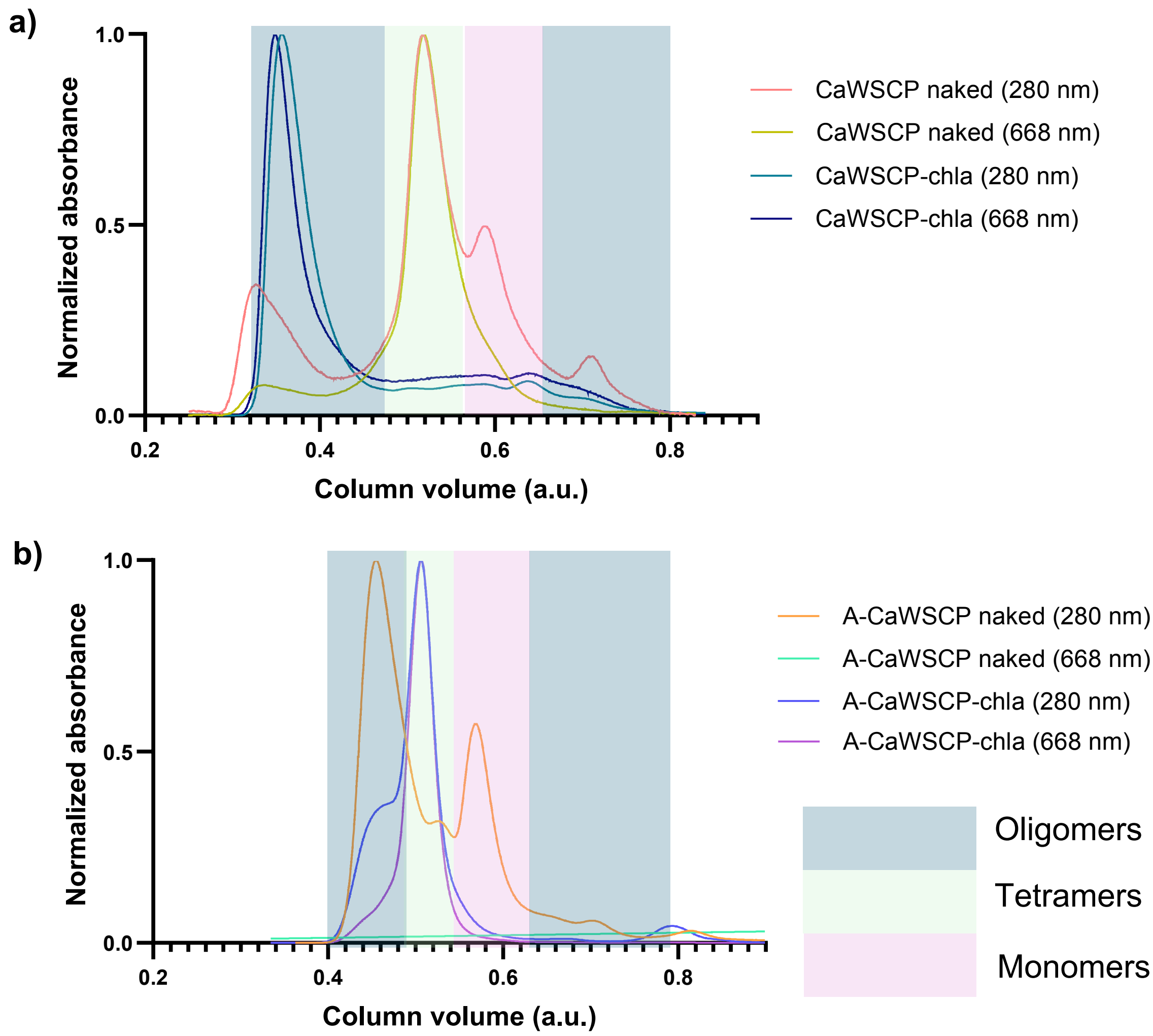

Supplementary Figure S1. Size exclusion chromatography (SEC) profiles comparing folding characteristics and tetramer formation of CaWSCP variants. a) CaWSCP (original version without SpyTag) shows increased aggregation and suboptimal folding properties. b) A-CaWSCP (engineered with SpyTag) demonstrates enhanced protein folding and reduced aggregation. The chromatograms display elution profiles where background coloured bands indicate distinct separation regions corresponding to different oligomeric states, with the tetramer form (desired for chlorophyll binding) clearly visible. Both panels compare naked proteins (without chlorophyll) and chlorophyll a (Chla)-bound proteins, revealing that A-CaWSCP exhibits superior folding properties and tetramer formation compared to standard CaWSCP. Naked proteins show folding challenges, while Chla binding promotes the desired tetrameric conformation. SEC was employed post-purification to isolate only the properly folded tetrameric fraction for subsequent experiments.

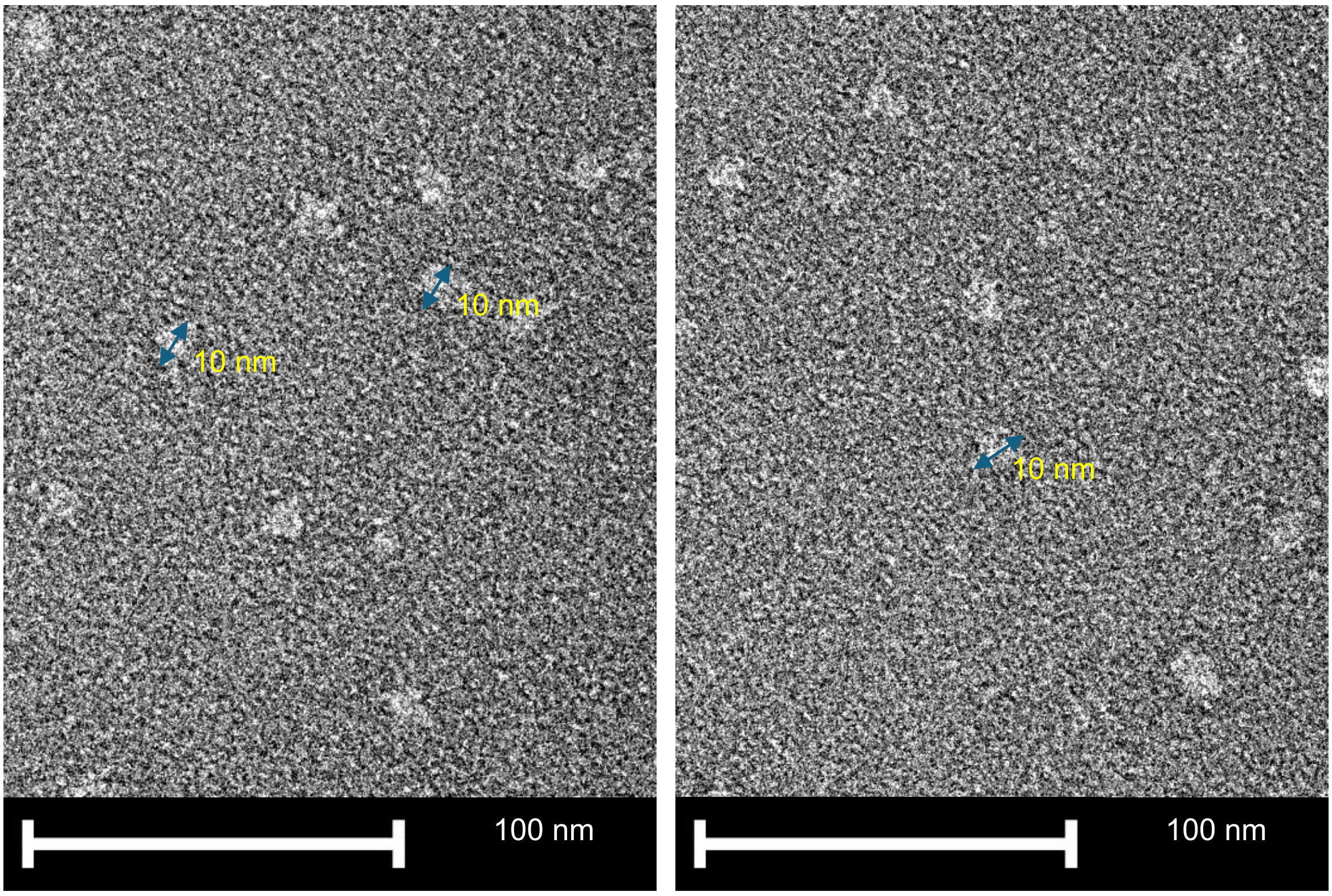

Supplementary Figure S2. Transmission electron microscopy (TEM) characterization of purified A-CaWSCP tetramers. The TEM micrographs display homogeneous A-CaWSCP protein complexes following purification and SEC. Representative tetrameric structures (indicated by blue arrows) exhibit consistent dimensions of approximately 10 nm, confirming the successful isolation of properly folded A-CaWSCP. The uniform morphology and size distribution throughout the field of view demonstrate the high purity and structural homogeneity achieved through the optimized purification protocol.

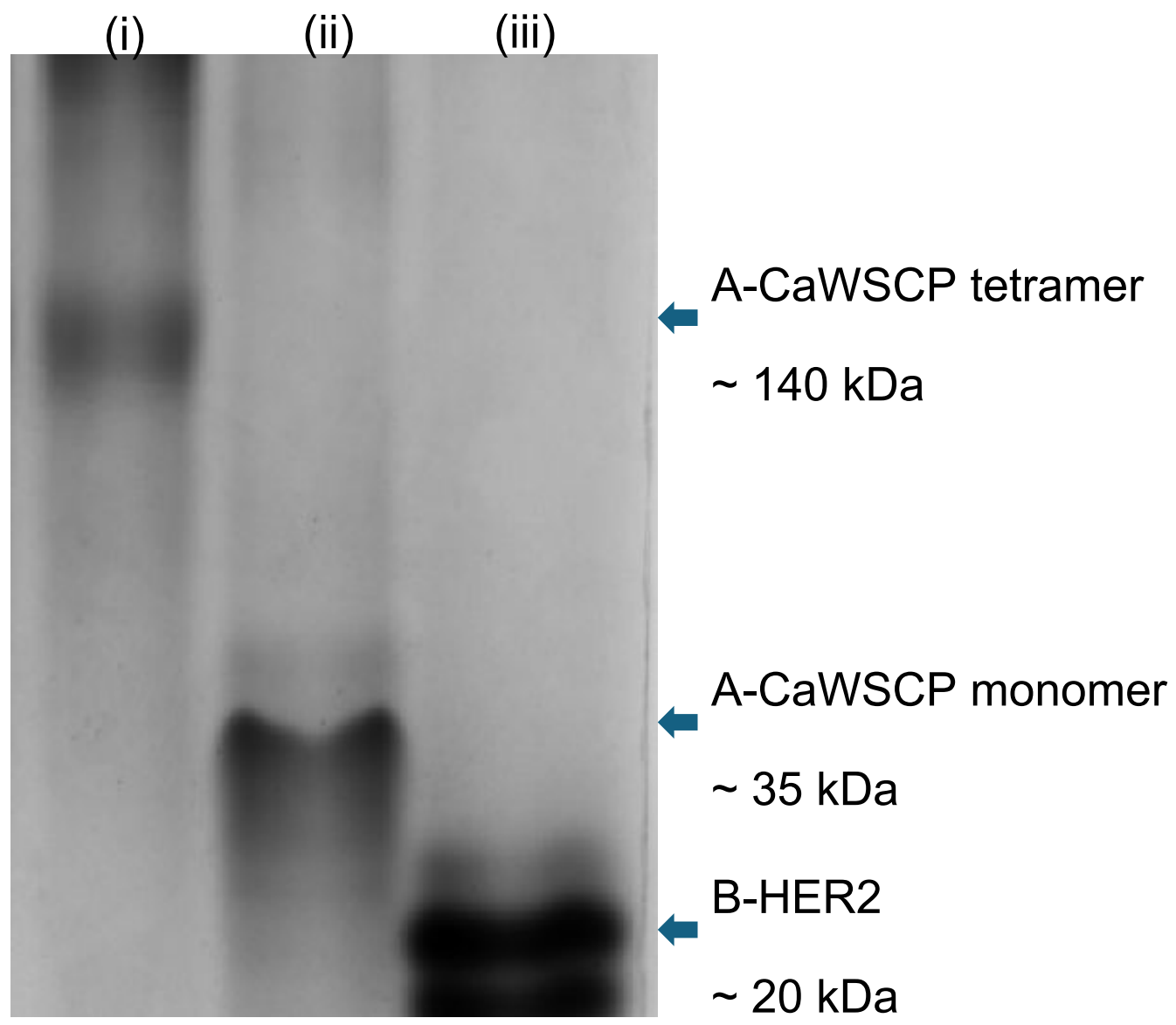

Supplementary Figure S3. Native PAGE analysis confirming homogeneity and oligomeric state of purified A-CaWSCP. The gel electrophoresis results demonstrate the purity and structural integrity of A-CaWSCP following SEC. Lane comparison shows distinct bands for: (i) A-CaWSCP tetramers purified by SEC (chlorophyll-bound form), (ii) A-CaWSCP monomers without chlorophyll, and (iii) B-HER2 reference protein. The sharp, well-defined band of the A-CaWSCP tetramer confirms successful purification and the absence of significant impurities or degradation products. This analysis corroborates the homogeneity of the A-CaWSCP preparation previously visualized by TEM in Supplementary Figure S2.

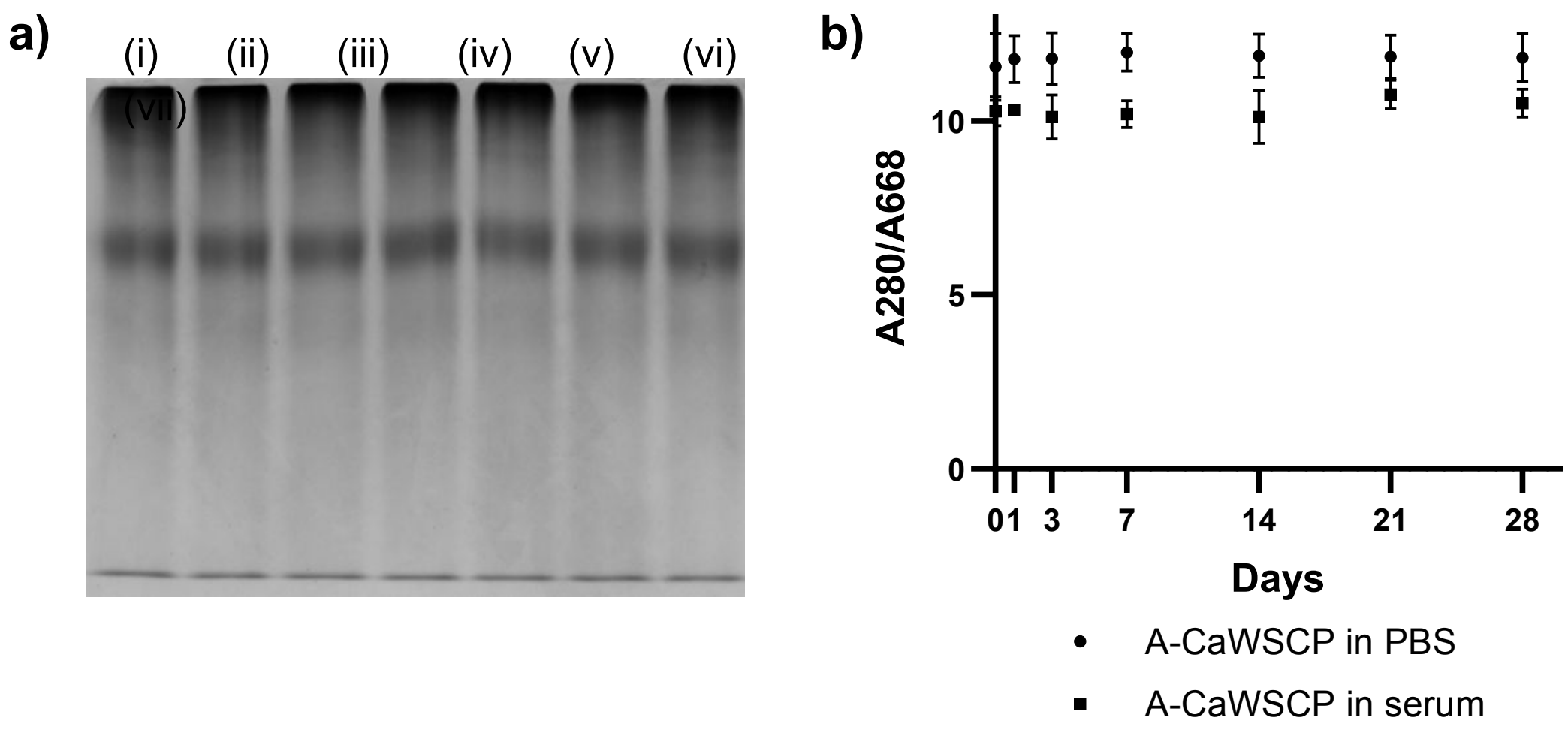

Supplementary Figure S4. Long-term stability assessment of A-CaWSCP in serum and PBS. a) Native PAGE analysis of A-CaWSCP tetramers in serum over 28 days showing bands at: (i) day 0, (ii) day 1, (iii) day 3, (iv) day 7, (v) day 14, (vi) day 21, and (vii) day 28. The consistent band intensity and position demonstrate remarkable structural stability of the tetrameric complex without significant degradation throughout the extended incubation period. b) Quantitative analysis comparing the ratio of protein absorbance (280 nm) to chlorophyll absorbance (668 nm) for A-CaWSCP in both serum and PBS over 28 days. One-way ANOVA with Tukey's post-hoc test revealed no statistically significant differences in absorbance ratios across all time points ($p > 0.05$), confirming that both protein integrity and chlorophyll binding capacity remain stable under physiological conditions.

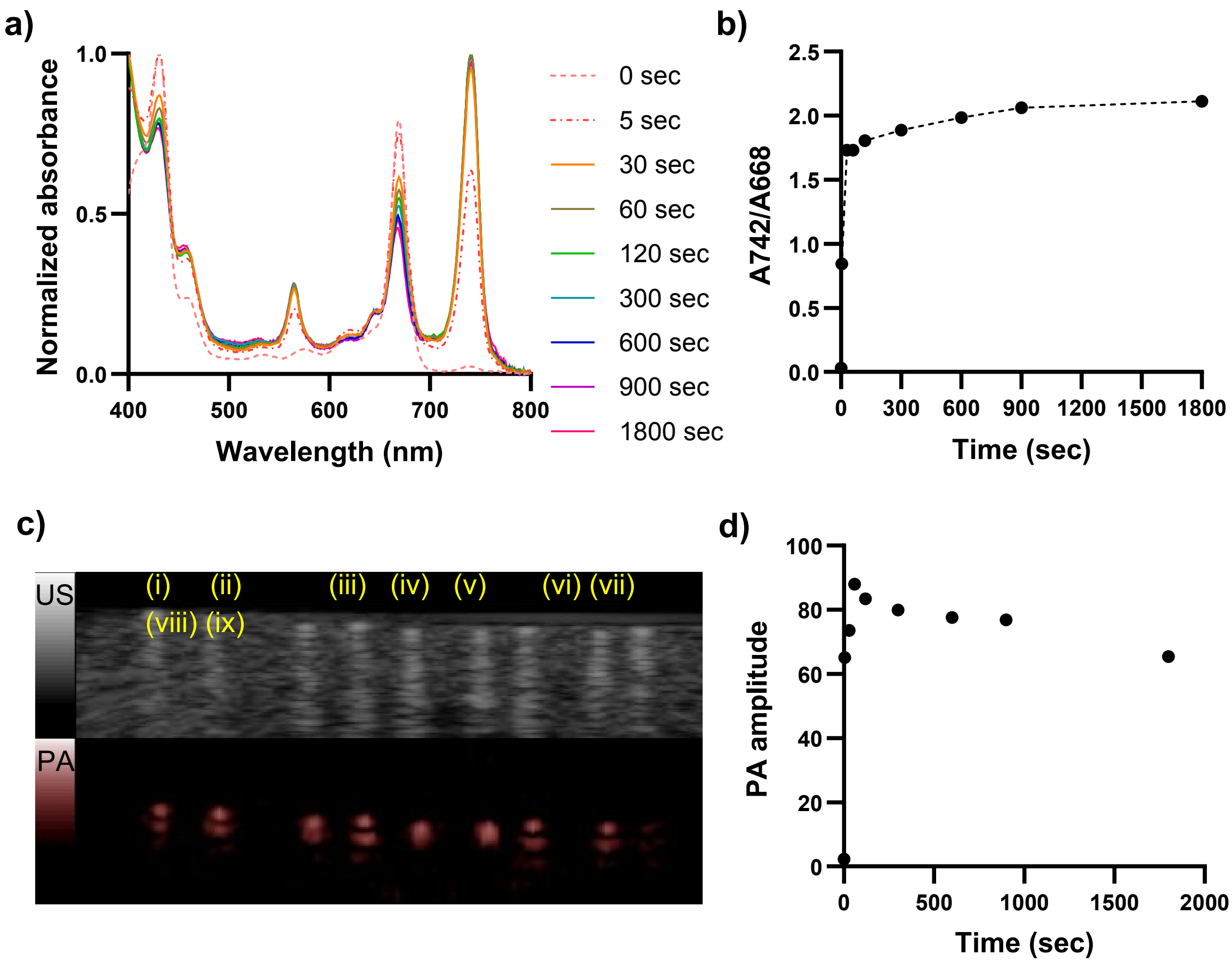

Supplementary Figure S5. Photoconversion kinetics and photoacoustic stability of A-CaWSCP under prolonged light exposure. a) Absorbance spectra of A-CaWSCP recorded at various time points from 0 seconds to 30 minutes of light exposure, demonstrating the gradual spectral shift from 668 nm to 742 nm during photoconversion. b) Quantitative analysis of the 742 nm/668 nm absorbance ratio over time, showing the photoconversion process reaching a plateau, indicating near-complete conversion of the chlorophyll-protein complex. c) Photoacoustic (PA) signal measurements at 742 nm in glass capillary phantoms [(i) 0 sec through (ix) 1800 sec] during continuous illumination. d) Plot of PA signal intensity at 742 nm versus illumination time in phantom tests, demonstrating remarkable photostability with no significant photobleaching observed throughout the 30-minute exposure period.

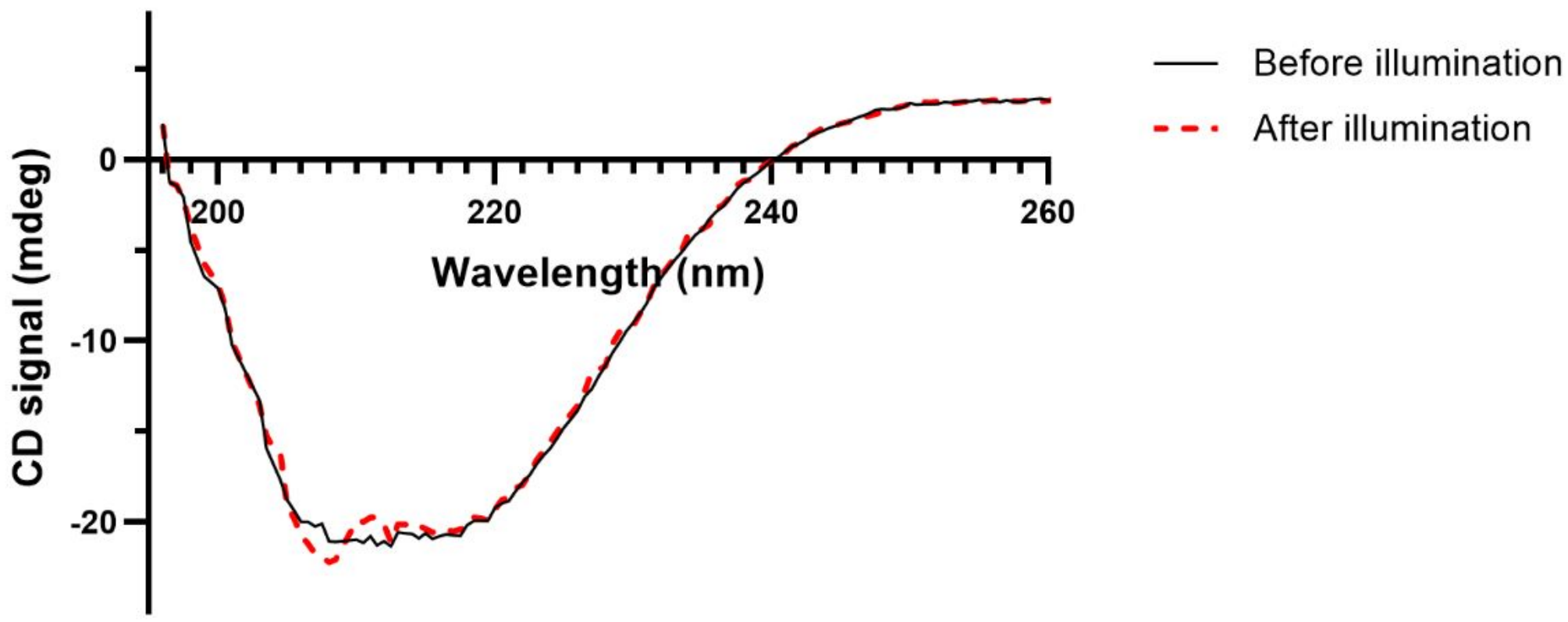

Supplementary Figure S6. Far-UV circular dichroism (CD) analysis of A-CaWSCP before and after photoconversion. Far-UV CD spectra of A-CaWSCP recorded before and after 668 nm illumination (photoconversion from 668 nm to 742 nm) exhibit highly similar spectral profiles, with preserved characteristic features in the 195–260 nm region. These results indicate that photoconversion does not induce detectable disruption of the protein's secondary structure, supporting the structural integrity of A-CaWSCP following illumination.

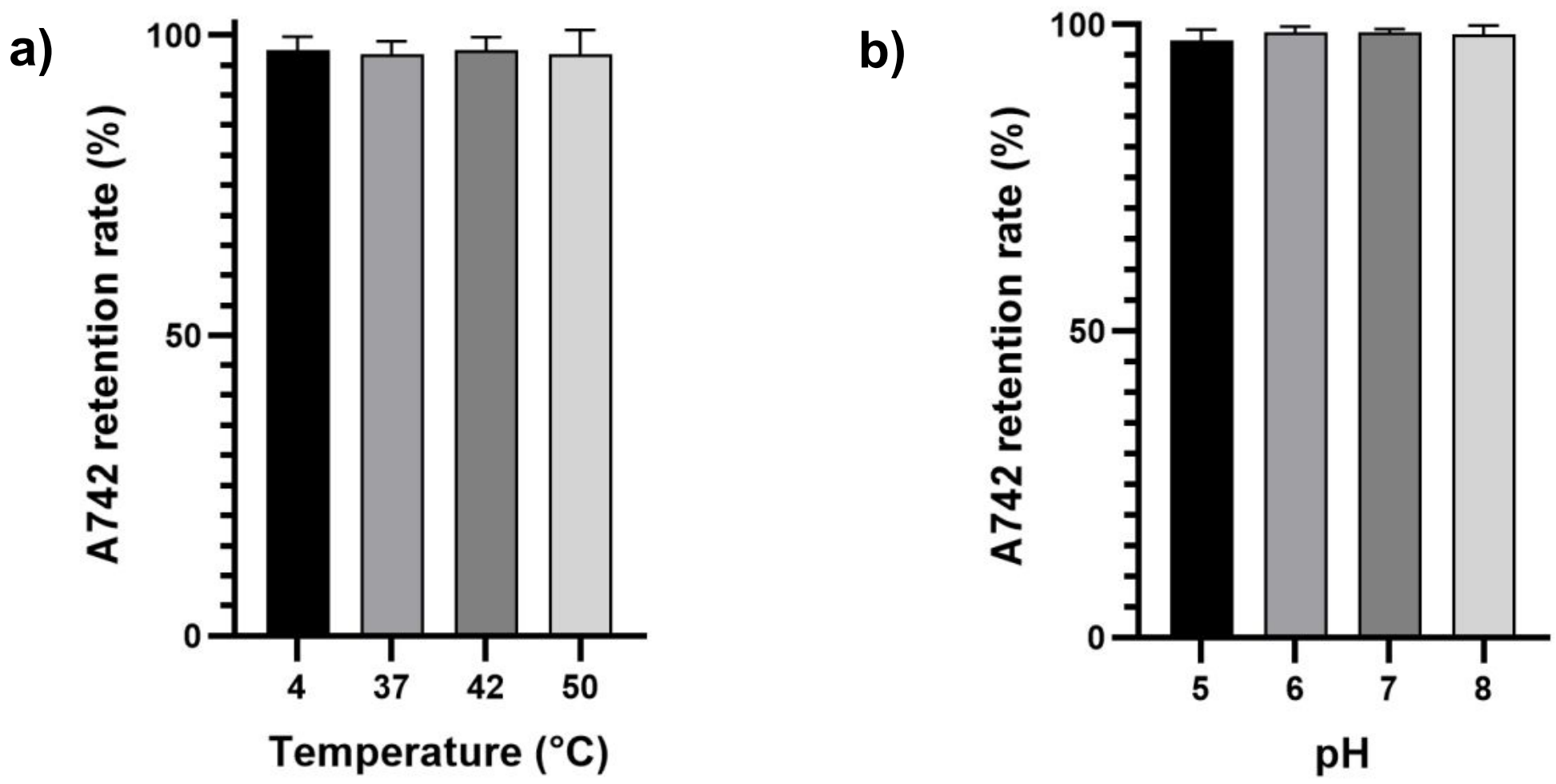

Supplementary Figure S7. Absorbance stability of the photoconverted A-CaWSCP state. (a) Retention of the 742 nm absorbance (A742) of photoconverted A-CaWSCP after 24 hour incubation at different temperatures (4-50°C) (b) Retention of A742 after 24 hour incubation under varying pH conditions (pH 5–8). Values are normalized to the absorbance immediately after photoconversion (0 hour). The photoconverted state remains highly stable (>90% retention) across all tested conditions, supporting its suitability for PA imaging workflows.

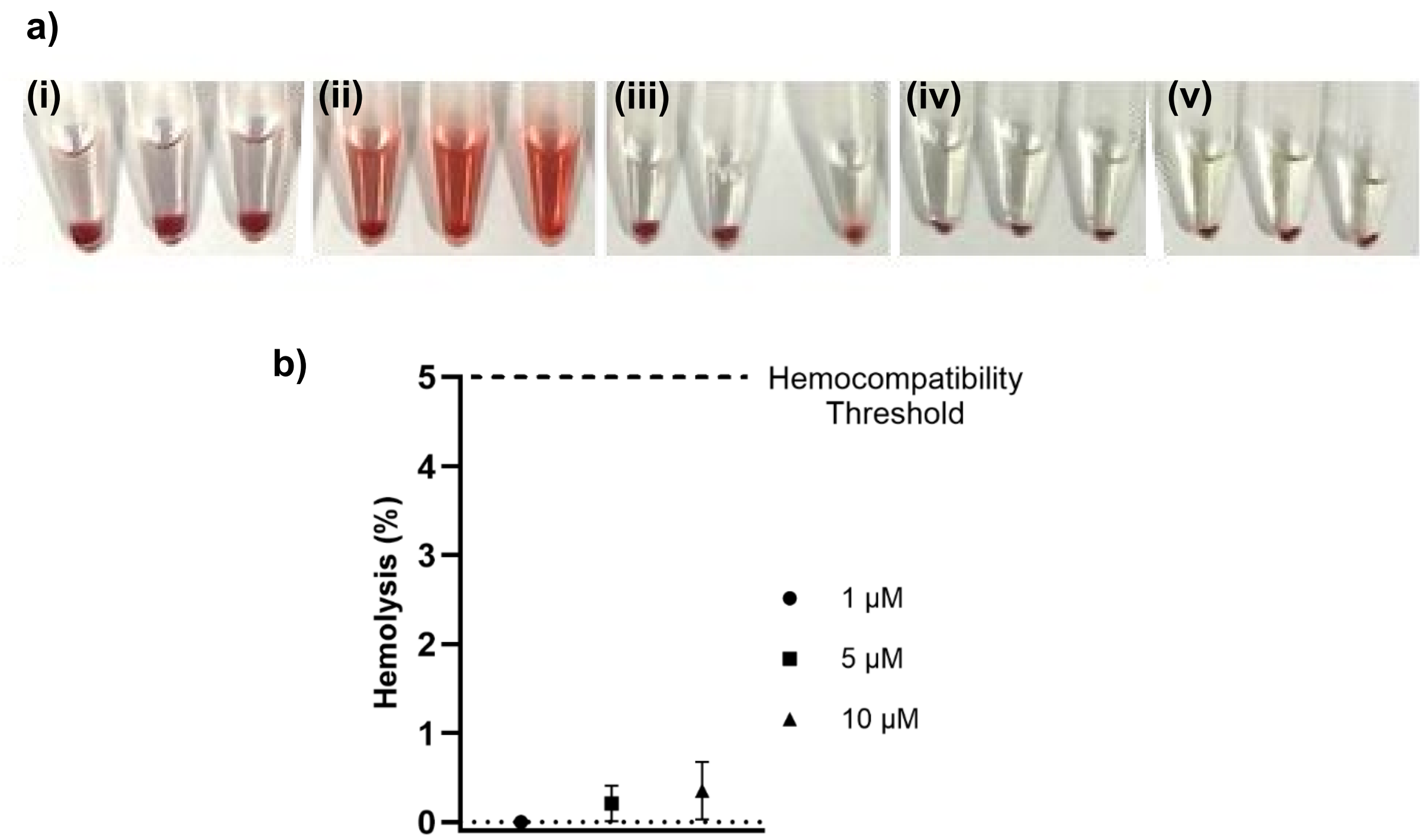

Supplementary Figure S8. Hemocompatibility assessment of CaWSCP-AB-HER2. (a) Representative photographs of red blood cell suspensions following incubation with (i) PBS (negative control), (ii) deionized water (positive control), and A-CaWSCP at (iii) 1 µM, (iv) 5 µM, and (v) 10 µM. PBS-treated samples show intact red blood cell pellets, whereas water-treated samples exhibit complete hemolysis. A-CaWSCP-treated samples display no visible hemolysis. (b) Quantitative analysis of hemolysis percentage, calculated relative to water-treated samples (100% hemolysis). A-CaWSCP exhibits negligible hemolytic activity (<5%) across all tested concentrations, indicating good hemocompatibility.

a)

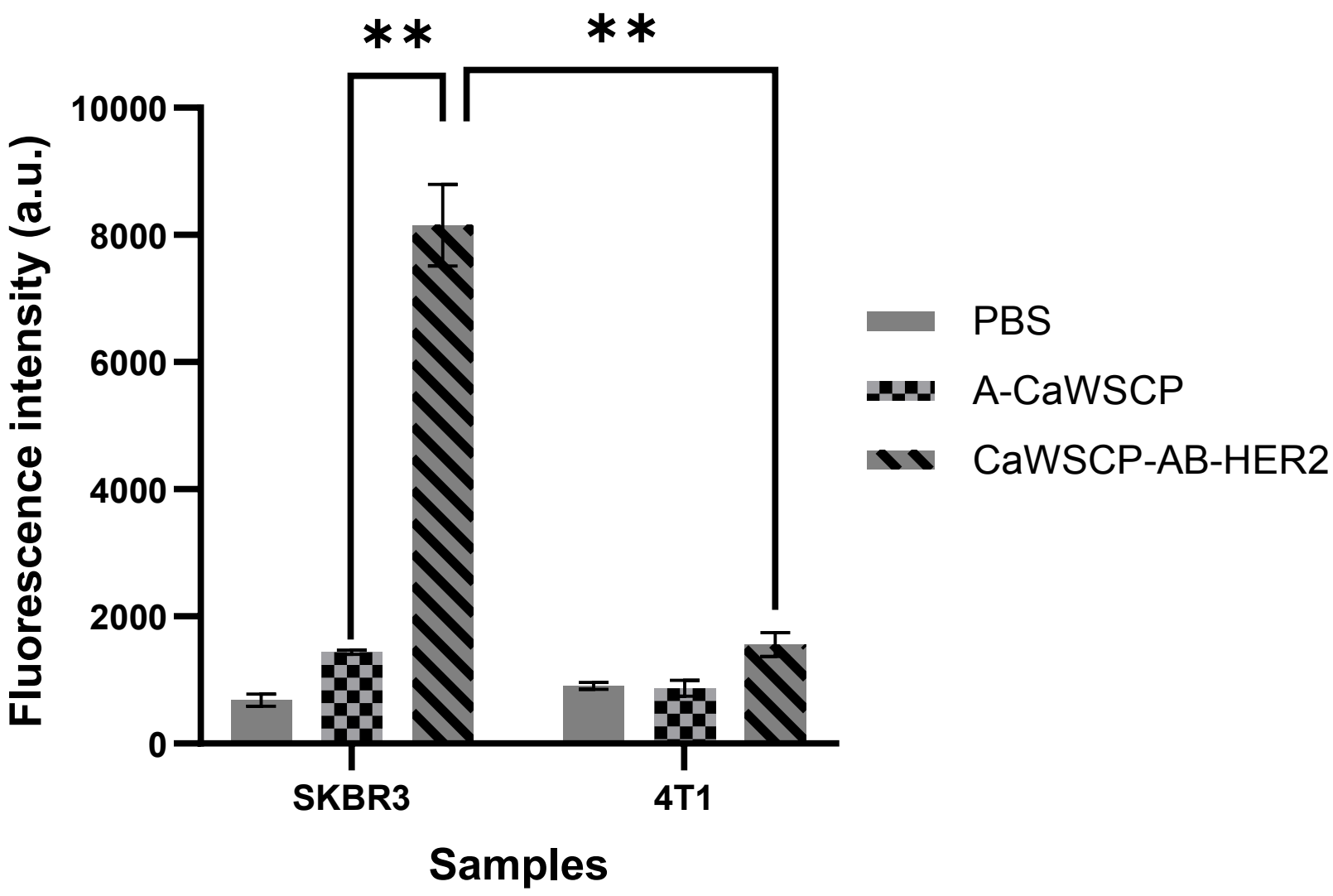

b)

		Fold differences	Cohen's d
SKBR3	SKBR3 A-CaWSCP	1.94	23.26
SKBR3	SKBR3 CaWSCP-AB-HER2	11.58	62.01
SKBR3 A-CaWSCP	SKBR3 CaWSCP-AB-HER2	5.96	55.73
4T1	4T1 A-CaWSCP	1.01	0.29
4T1	4T1 CaWSCP-AB-HER2	1.79	16.24
4T1 A-CaWSCP	4T1 CaWSCP-AB-HER2	1.77	16.75
SKBR3 CaWSCP-AB-HER2	4T1 CaWSCP-AB-HER2	5.14	53.08
SKBR3 A-CaWSCP	4T1 A-CaWSCP	1.52	13.52

Supplementary Figure S9. Quantitative flow cytometry analysis demonstrating selective targeting of HER2-positive cancer cells by CaWSCP-AB-HER2. a) Fluorescence intensity measurements comparing binding of different constructs to SKBR3 (HER2-positive) and 4T1 (HER2-negative) cell lines. CaWSCP-AB-HER2 exhibits dramatically enhanced binding to SKBR3 cells (11.58-fold increase compared to PBS control), while showing minimal non-specific binding to 4T1 cells. In contrast, non-targeted A-CaWSCP shows negligible binding to either cell line, confirming the specificity of the antibody-conjugated construct. b) Statistical analysis of binding interactions using fold differences and Cohen's d effect size measurements. The exceptionally high Cohen's d value for SKBR3 with CaWSCP-AB-HER2 (compared to controls) demonstrates the robust targeting capability and strong statistical significance of the observed binding preference.

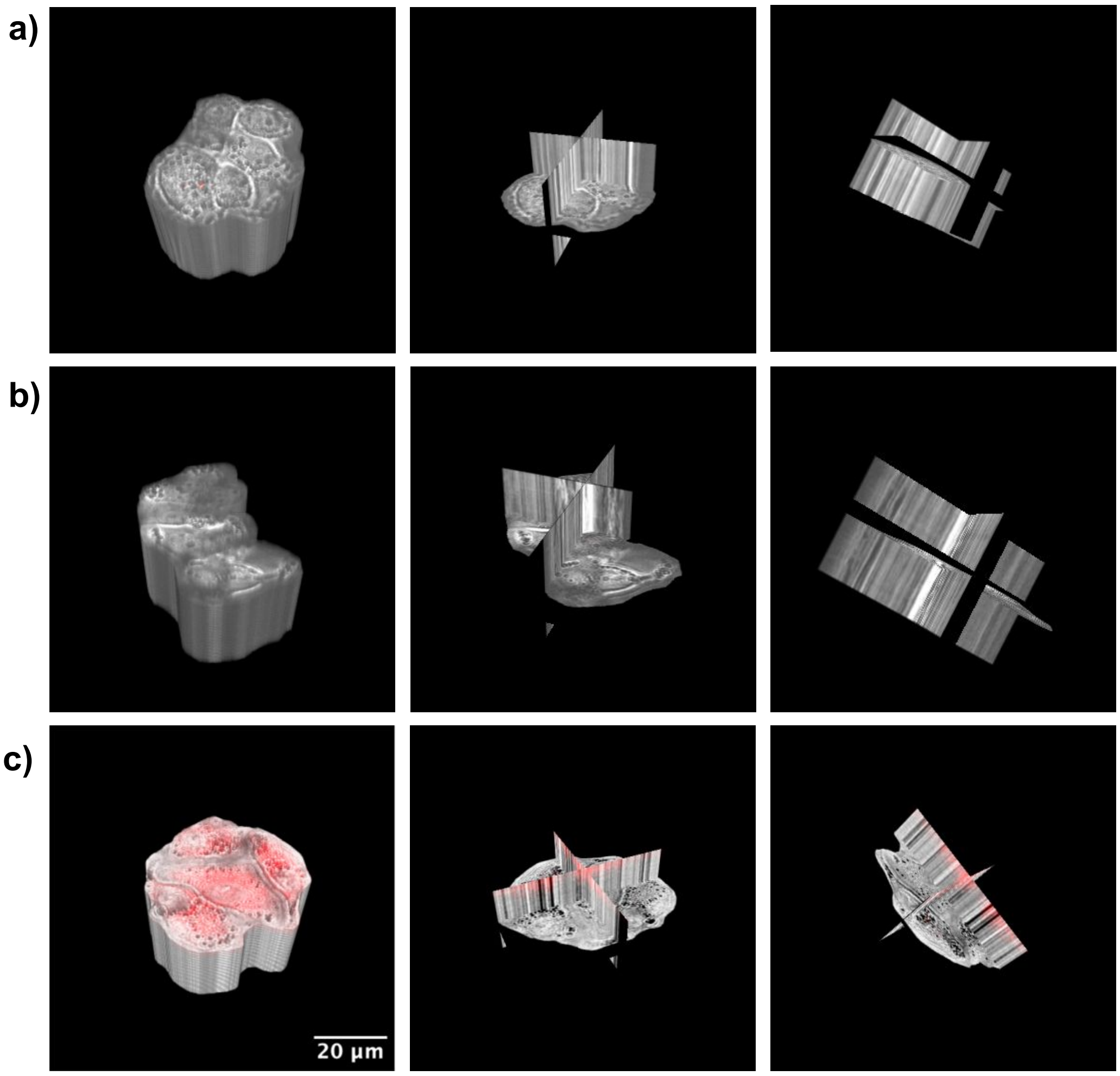

Supplementary Figure S10. Confocal microscopy analysis of cellular internalization and surface localization. Representative 3D reconstructions of SKBR3 cells following incubation with (a) PBS (blank), (b) A-CaWSCP (5 µM), and (c) CaWSCP-AB-HER2 (5 µM). For each condition, three views are shown: 3D volume rendering (left), orthogonal/side view (middle), and *xz* projection (right), reconstructed from *Z*-stack confocal images (99 slices). Brightfield and Cy5 fluorescence signals are merged for visualization. Blank and A-CaWSCP-treated cells exhibit minimal fluorescence, whereas CaWSCP-AB-HER2-treated cells display a pronounced cell-associated Cy5 signal with a predominantly peripheral distribution, consistent with HER2-mediated surface binding and limited intracellular accumulation under the tested conditions. Scale bar, 20 µm.

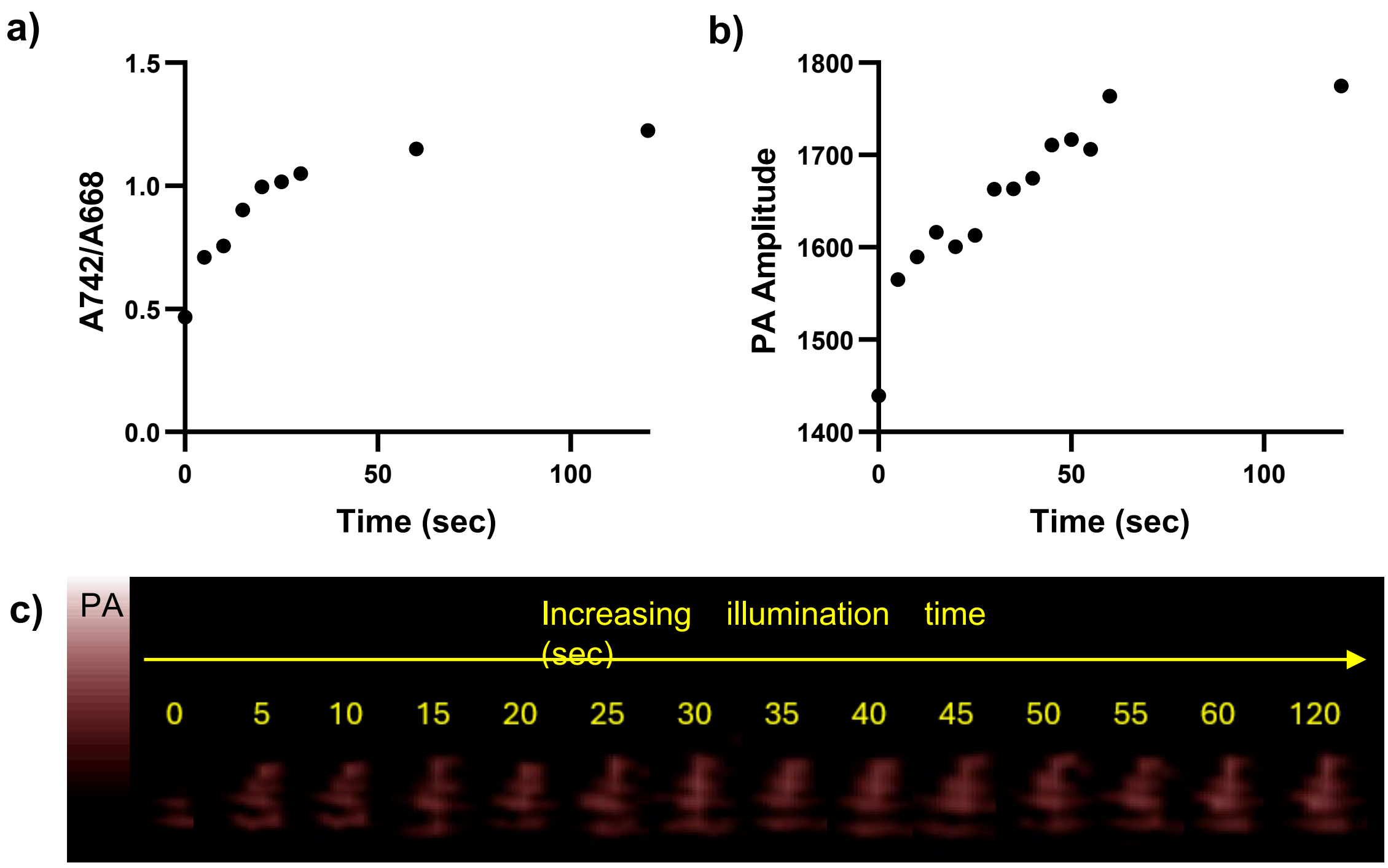

Supplementary Figure S11. Correlation between photoconversion and photoacoustic signal enhancement of A-CaWSCP. a) Spectral ratio (A742/A668) showing progressive photoconversion with increasing illumination time (0–120 seconds). b) Corresponding PA amplitude measurements revealing a direct relationship between photoconversion extent and PA signal. c) Representative PA images acquired at sequential time points, demonstrating enhancement of PA signal (red) that correlates with photoconversion progression, validating the mechanism of contrast enhancement through controlled photoconversion.

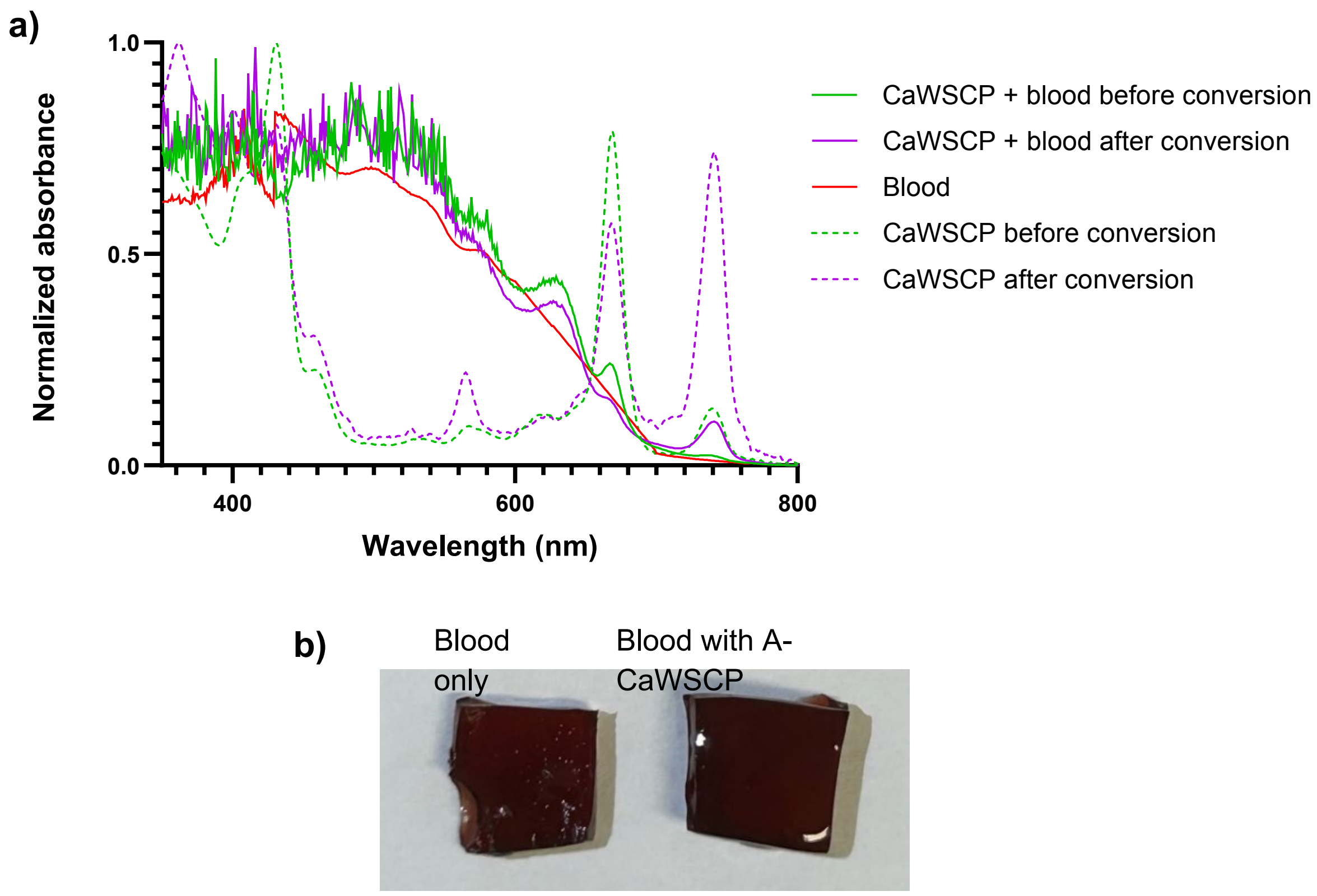

Supplementary Figure S12. Spectral analysis of A-CaWSCP in blood and visual assessment of detection challenges. a) Normalized absorbance spectra comparing blood only (red line), CaWSCP with blood before photoconversion (green line), CaWSCP with blood after photoconversion (pink line), pure CaWSCP before conversion (purple dotted line), and pure CaWSCP after conversion (green dotted line). The spectral overlap between blood and A-CaWSCP demonstrates the challenge of distinguishing the contrast agent signal from background blood absorption. b) Photographic comparison of blood-containing gelatine phantoms with and without A-CaWSCP, illustrating the visual similarity between samples that prevents differentiation by naked eye observation. These results highlight the necessity for differential imaging techniques to effectively isolate the A-CaWSCP signal from background absorption.

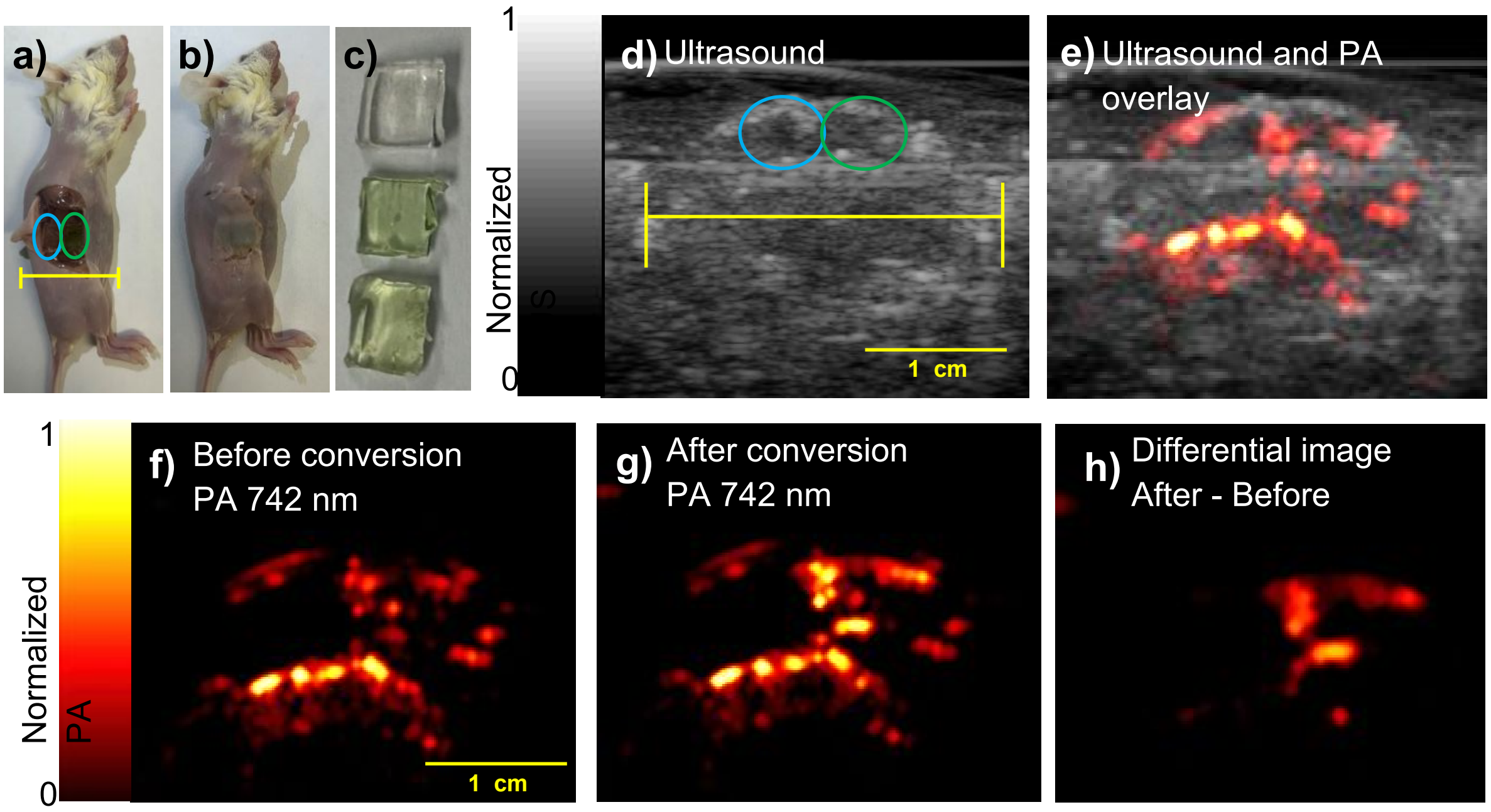

i)

	Weber contrast	PA amplitude
Before conversion – PBS region	0.143±0.032	
Before conversion – PBS+A-CaWSCP region	0.218±0.011	
After conversion – PBS region	0.127±0.034	
After conversion – PBS+A-CaWSCP region	1.194±0.081	
Differential – PBS region		1.622±0.052
Differential - PBS+A-CaWSCP region		28.53±1.23

Supplementary Figure S13. Ex vivo differential PA imaging validation in PBS-containing tissue-mimicking phantoms as preliminary validation before blood phantom experiments. a) Photograph of a mouse model with skin sealed back. b) Photograph of gelatin phantoms with imaging regions of interest. c) Visual comparison of gelatin phantoms containing PBS only (top), A-CaWSCP without photoconversion (middle), and A-CaWSCP after photoconversion (bottom), showing subtle color differences difficult to distinguish by eye. d) Ultrasound (US) image showing the structural information of phantoms. The interstitial domain that served as the background ROI is indicated by yellow lines. e) Combined US and PA overlay image before photoconversion. f) PA-only image before photoconversion at 742 nm. g) PA-only image after photoconversion at 742 nm. h) Differential image (after - before) demonstrating enhanced contrast in the A-CaWSCP region. i) Signal-to-Noise Ratio (SNR) measurements comparing PBS regions and PBS+A-CaWSCP regions before conversion, after conversion, and in differential images, highlighting the dramatic SNR improvement (3.920±0.139) achieved through the differential imaging approach. This PBS phantom validation served as a critical precursor to the more complex blood-containing phantom experiments presented in the main figure.

a) SpyTag-elp-SaCI-CaWSCP-SpeI-elp-SpyTag

b) AHIVMVDAYKPTKLDGHGVGVPGVGVPGVGVPGEGVPGVGVPGVGVPGVGVPGVGVPGE
GVPGVGVPGVGVPGVGVPGVGVPGEGVPGVGVPGVGELGSHCPASTIPDILKYYGLPPSIFP
GNVQSFSCDLVNKNSIRLTINLLGECTVVNELGAIQNVLKCSEKISAVLSHNKLTEVKGVTVQL
YVKLLPWVDAAPFGPLMTITGAEASHGPLNLKLFKFISDQGESPAFPYILFPNQAPKCTHLAFY
DTVVANTNTNLPFNPFLVATSVPGVGVPGVGVPGEGVPGVGVPGVGVPGVGVPGVGVPGE
GVPGVGVPGVGVPGVGVPGVGVPGEGVPGVGVPGVGVPGGLLDAHIVMVDAYKPTKLD-LE

Supplementary Figure S14. Molecular design and amino acid sequence of the A-CaWSCP construct. a) Schematic representation of the engineered A-CaWSCP fusion protein architecture, featuring SpyTag domains at both termini flanking elastin-like polypeptide (ELP) sequences and the core CaWSCP protein. b) Complete amino acid sequence of the A-CaWSCP construct. The sequence includes SpyTag domains for site-specific conjugation, elastin-like polypeptide (ELP) repeats (VGVPG motifs) for enhanced solubility and stability, and the chlorophyll-binding CaWSCP domain. This engineered construct enables both efficient chlorophyll binding for photoacoustic imaging and specific antibody conjugation for targeted molecular imaging applications.

a) SpyCatcher-HER2affibody

b) HMHHHHHHAMVDTLSGLSSEQGQSGDMTIEEDSATHIKFSKRDEDGKELAGATMELRDS
SGKTISTWISDGQVKDFYLYPGKYTFVETAAPDGYEVATAITFTVNEQGQVTVNGKATKG
DAHIDGPQGIWGQLDGRGVDNKFNKEMRNAYWEIALLPNLNNQQKRAFIRSLYDDPSQS
ANLLAEAKKLNDAQAPK-LE

Supplementary Figure S15. Molecular design and amino acid sequence of the B-HER2 targeting construct. a) Schematic representation of the engineered B-HER2 fusion protein architecture, featuring a histidine tag (His-tag) for purification, SpyCatcher domain for conjugation to A-CaWSCP, and HER2-targeting affibody for specific cancer cell recognition. b) Complete amino acid sequence of the B-HER2 construct. The sequence begins with the histidine tag (HMHHHHH), followed by the SpyCatcher domain for covalent binding to the SpyTag domains of A-CaWSCP, and the HER2-targeting affibody domain that provides specific binding to HER2-overexpressing cancer cells.

a) SpyTag-elp-SaCI-eGFP-SpeI-elp-SpyTag

b) AHIVMVDAYKPTKLDGHGVGVPGVGVPGVGVPGEGVPGVGVPGVGVPGVGVPGVGVPGE
GVPGVGVPGVGVPGVGVPGVGVPGEGVPGVGVPGVGELMVSKGEELFTGVVPILVELDGD
VNGHKFSVSGEGEGDATYGKLTLKFICTTGKLPVPWPTLVTTLTYGVQCFSRYPDHMKQHD
FFKSAMPEGYVQERTIFFKDDGNYKTRAEVKFEGDTLVNRIELKGIDFKEDGNILGHKLEYNY
NSHNVYIMADKQKNGIKVNFKIRHNIEDGSVQLADHYQQNTPIGDGPVLLPDNHYLSTQSALS
KDPNEKRDHMVLLEFVTAAGITLGMDELYK*TSVPGVGVPGVGVPGEGVPGVGVPGVGVPG
VGVPGVGVPGEGVPGVGVPGVGVPGVGVPGVGVPGEGVPGVGVPGVGVPGGLLDAHIVM
VDA

c)

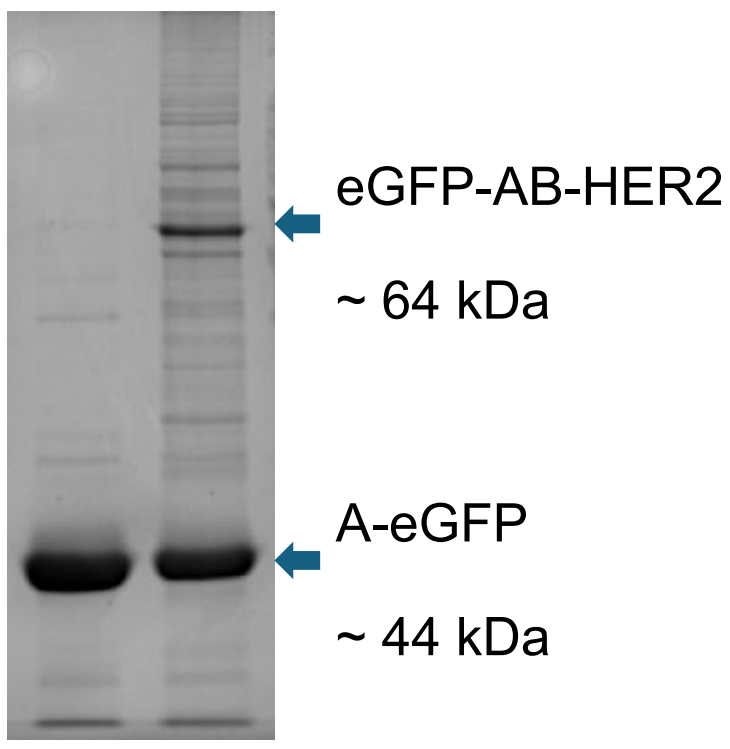

Supplementary Figure S16. Molecular design, amino acid sequence, and expression validation of the A-eGFP control construct. a) Schematic representation of the engineered A-eGFP fusion protein architecture, featuring the same structural framework as A-CaWSCP but with eGFP substituted for the CaWSCP domain. The construct maintains identical SpyTag domains at both termini flanking elastin-like polypeptide (ELP) sequences surrounding the central eGFP protein. b) Complete amino acid sequence of the A-eGFP construct. This control protein follows the same design principles as A-CaWSCP, enabling direct comparison of functionality while utilizing the commercially available enhanced Green Fluorescent Protein (eGFP) in place of CaWSCP. c) SDS-PAGE analysis confirming successful expression and purification of A-eGFP (44 kDa, left lane) and eGFP-AB-HER2 conjugate (64 kDa, right lane). The distinct bands at the expected molecular weights validate the integrity of both the individual A-eGFP construct and its successful conjugation with the B-HER2 targeting module.

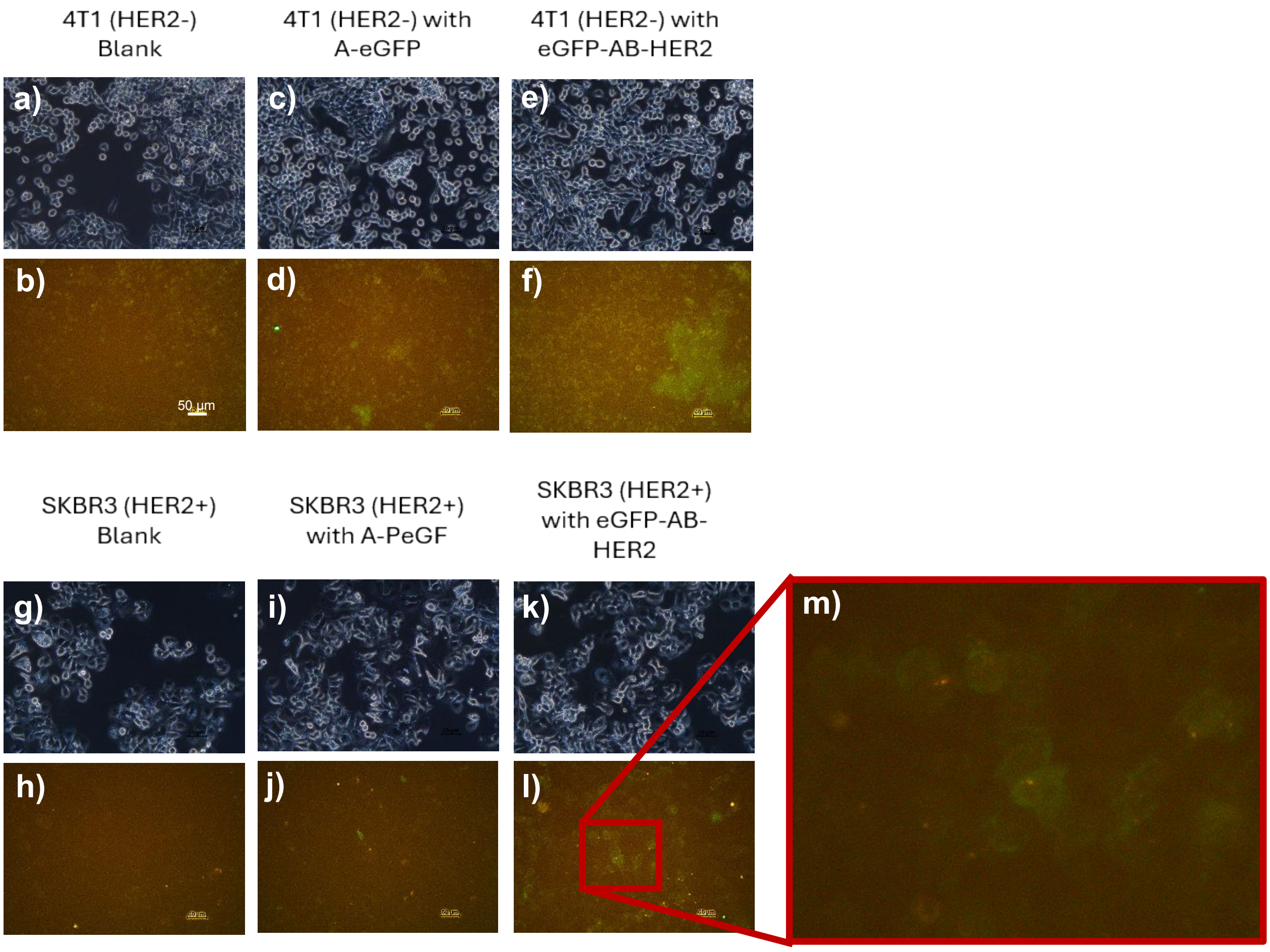

Supplementary Figure S17. Validation of HER2-targeting specificity using fluorescent protein constructs. The panel shows binding patterns of A-eGFP and eGFP-AB-HER2 to HER2-negative (4T1) and HER2-positive (SKBR3) cell lines. Top row: 4T1 (HER2-) cells with (a,b) blank control, (c,d) A-eGFP, and (e,f) eGFP-AB-HER2. Bottom row: SKBR3 (HER2+) cells with (g,h) blank control, (i,j) A-eGFP, and (k,l) eGFP-AB-HER2. For each condition, brightfield images (a,c,e,g,i,k) are paired with corresponding fluorescence images (b,d,f,h,j,l). 4T1 cells show only random, non-specific binding of A-eGFP (green smudges). In contrast, SKBR3 cells treated with eGFP-AB-HER2 display distinct circular fluorescence patterns around cell membranes, indicating specific binding to HER2 receptors. Panel (m) provides a magnified view of this membrane-localized binding pattern. These results confirm the functionality of the SpyTag/SpyCatcher conjugation system and validate the HER2-targeting capability of the affibody construct, supporting the design approach used for the CaWSCP-based imaging agent.

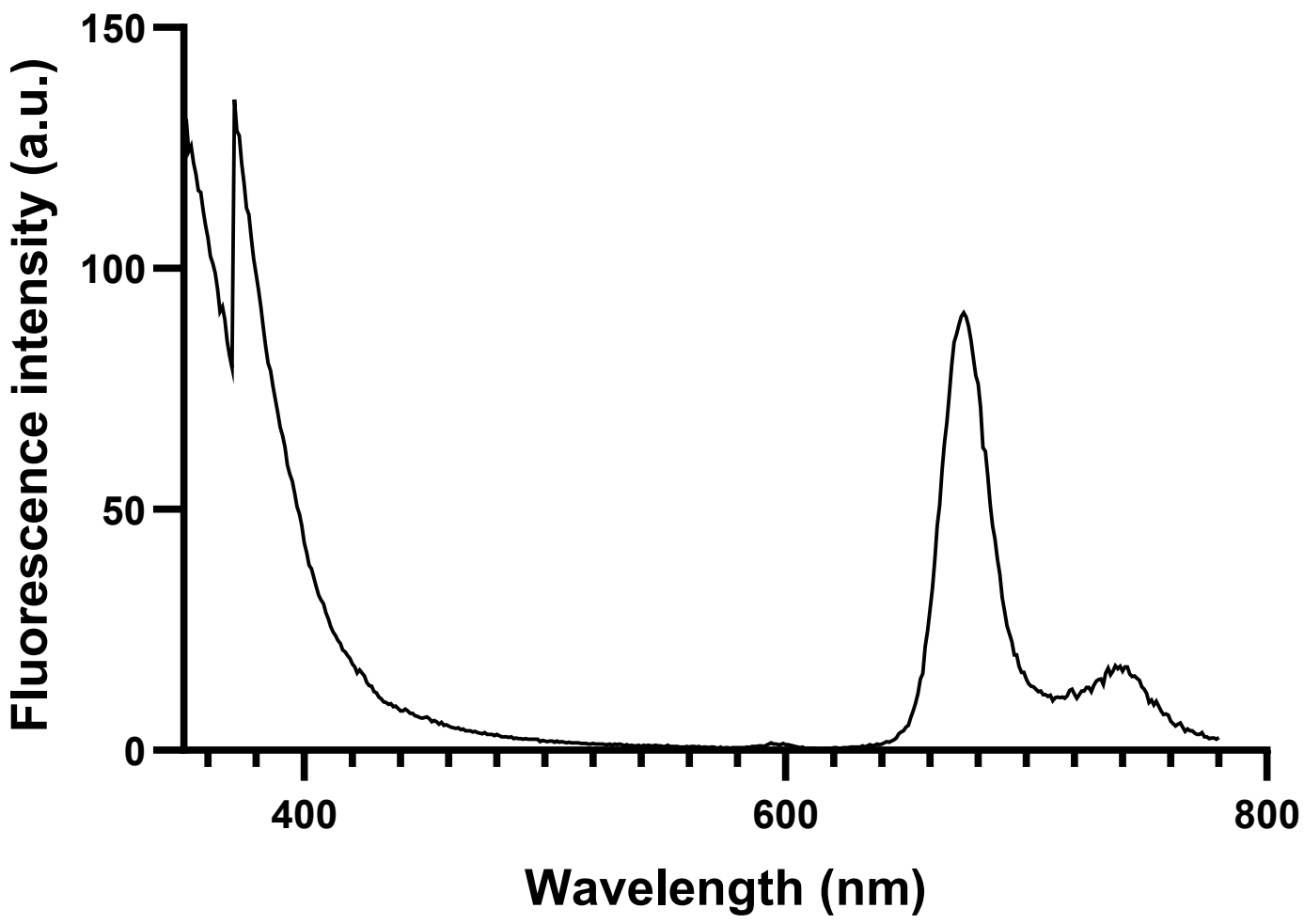

Supplementary Figure S18. Fluorescence emission spectrum of A-CaWSCP. The spectrum demonstrates the modest but detectable fluorescence properties of the A-CaWSCP construct. While the fluorescence intensity is relatively low compared to dedicated fluorescent proteins, the measurable emission signal proved sufficient for complementary analytical techniques, including flow cytometry and confocal microscopy imaging. This inherent fluorescence capability provided an additional validation method for cellular binding studies and localization experiments, complementing the primary photoacoustic imaging applications of the contrast agent.

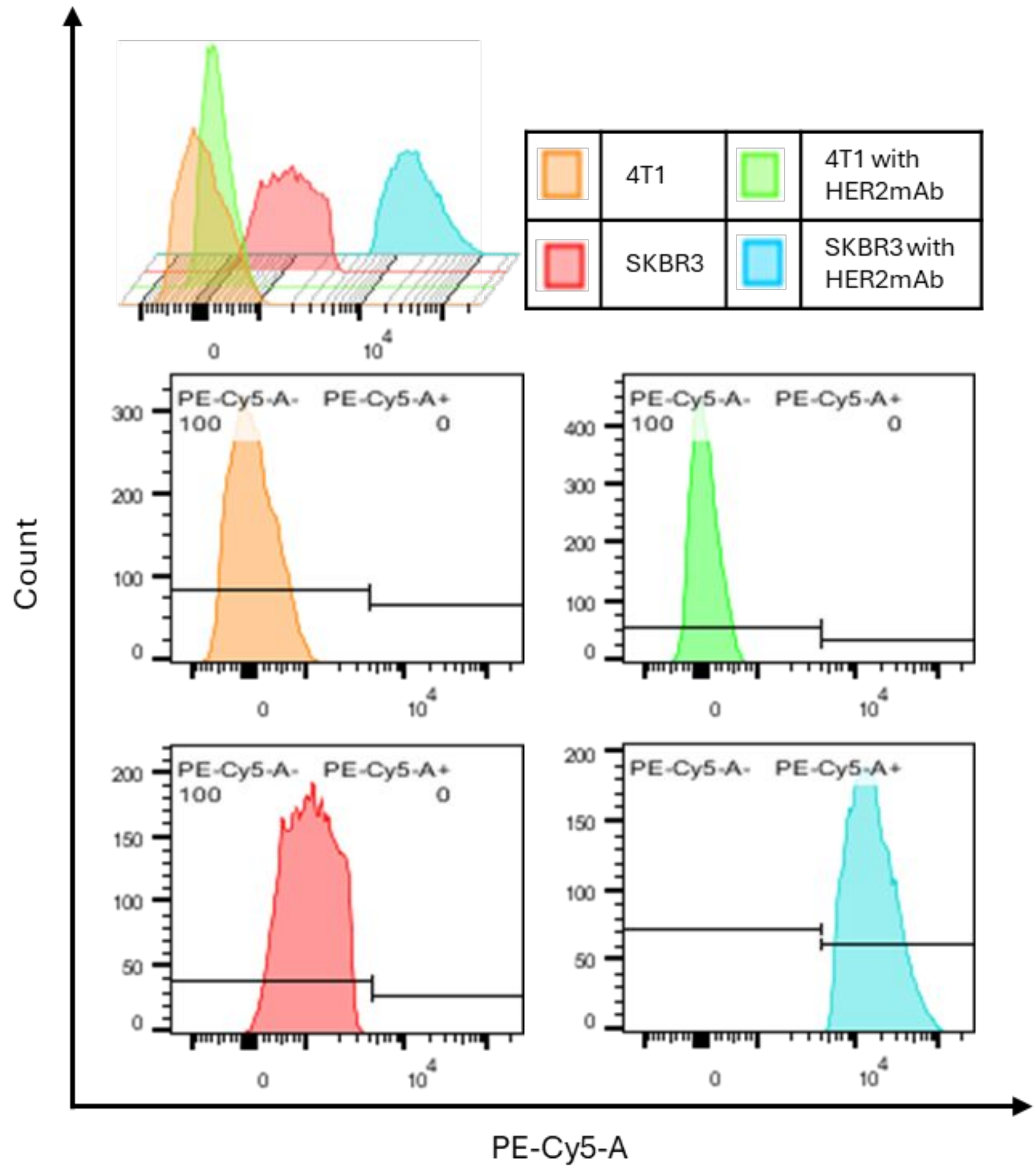

Supplementary Figure S19. Flow cytometry validation of HER2 expression levels in experimental cell lines. The figure shows comparative flow cytometry analysis of HER2 surface expression in 4T1 (HER2-negative) and SKBR3 (HER2-positive) cell lines. The main histogram (top left) displays overlaid fluorescence intensity distributions for anti-HER2 antibody binding across different cell populations, with distinct peaks representing varying levels of HER2 expression. Individual histograms (bottom panels) show separate fluorescence profiles for each experimental condition. The clear difference in fluorescence intensity between SKBR3 and 4T1 cells confirms the appropriate HER2 expression status of the cell lines used in targeting experiments. This validation ensures that the observed differential binding of CaWSCP-AB-HER2 constructs is specifically due to HER2 targeting rather than non-specific interactions.
